# Biology and Clinical Management of Myeloproliferative Neoplasms and Development of the JAK Inhibitor Ruxolitinib

**DOI:** 10.2174/092986712803251511

**Published:** 2012-09

**Authors:** J Mascarenhas, TI Mughal, S Verstovsek

**Affiliations:** 1 Mount Sinai School of Medicine, New York, USA; 2 Tufts University School of Medicine, Boston, Massachusetts, USA; 3 The University of Texas MD Anderson Cancer Center, Houston, Texas, USA

**Keywords:** Essential thrombocythemia, janus kinase, JAK inhibitor, JAK-STAT, myelofibrosis, myeloproliferative neoplasms, polycythemia vera, primary myelofibrosis, quality of life, ruxolitinib, splenomegaly, symptoms.

## Abstract

Myeloproliferative neoplasms (MPN) are debilitating stem cell-derived clonal myeloid malignancies. Conventional treatments for the BCR-ABL1-negative MPN including polycythemia vera (PV), essential thrombocythemia (ET), and primary myelofibrosis (PMF) have, so far, been unsatisfactory. Following the discovery of dysregulated JAK-STAT signaling in patients with MPN, many efforts have been directed toward the development of molecularly targeted therapies, including inhibitors of JAK1 and JAK2. Ruxolitinib (previously known as INCB018424; Incyte Corporation, Wilmington, Delaware, USA) is a rationally designed potent oral JAK1 and JAK2 inhibitor that has undergone clinical trials in patients with PV, ET, and PMF. Ruxolitinib was approved on November 16, 2011 by the United States Food and Drug Administration for the treatment of intermediate or high-risk myelofibrosis (MF), including patients with PMF, post-PV MF, and post-ET MF. In randomized phase III studies, ruxolitinib treatment resulted in significant and durable reductions in splenomegaly and improvements in disease-related symptoms in patients with MF compared with placebo or best available therapy. The most common adverse events were anemia and thrombocytopenia, which were manageable and rarely led to discontinuation. This review addresses the cellular and molecular biology, and the clinical management of MPN.

## THE CLASSIFICATION, PATHOPHYSIOLOGY, AND SYMPTOMATOLOGY OF MYELOPROLIFERATIVE NEOPLASMS

### Classification

Myeloproliferative neoplasms (MPN) are stem cell-derived clonal myeloid malignancies [1]. The World Health Organization (WHO) classification system for hematopoietic malignancies recognizes MPN as a separate category which includes subcategories, based on diverse clinical, morphologic, and molecular findings. These subcategories are: polycythemia vera (PV), essential thrombocythemia (ET), primary myelofibrosis (PMF), chronic myelogenous leukemia (CML), chronic neutrophilic leukemia, chronic eosinophilic leukemia not otherwise specified, hypereosinophilic syndrome, mastocytosis, and MPN unclassifiable (Table **1**). In recognition of William Dameshek’s remarkable insight over six decades ago, when he collectively described PV, ET, PMF, and CML as ‘myeloproliferative disorders’ (MPD), these four subcategories are also referred to as ‘classic’ MPN. The term ‘*BCR-ABL1*-negative’ MPN, used interchangeably with the term ‘Philadelphia chromosome-negative’ MPN, refers to PV, ET, and PMF. The term myelofibrosis (MF) is used to describe PMF as well as the phenotypically-related conditions that can arise in patients with PV (termed post-PV MF; PPV-MF) or ET (termed post-ET MF; PET-MF). MPN are potentially debilitating malignant disorders that clearly require accurate diagnoses and risk stratification to help optimize management [2, 3].

In 2005, the discovery of an acquired mutation in Janus kinase 2 (*JAK2*), a cytoplasmic tyrosine kinase (TK), termed *JAK2*^V617F^, in patients with PV and related MPN, provided a unique opportunity for the re-evaluation of the diagnostic criteria describing MPN [4–8]. Sensitive methods demonstrate the presence of this mutation in more than 95% of patients with PV and over 50% of those with ET and PMF [9]. Accordingly, in 2008, WHO diagnostic criteria for PV, ET, and PMF were revised [2, 3]. Furthermore, these seminal observations ushered the era of targeting JAK2 on the notion of developing new treatments for patients with MPN [10]. 

Currently, there are several somatic MPN-associated mutations other than the seminal *JAK2*^V617F^ which have been reported in patients with MPN (Table **2**). These include mutations of the following genes:* MPL, TET2, ASXL1, IDH1, IDH2, CBL, IKZF1, LNK, *and *EZH2 *which appear to originate at the stem cell (progenitor) level; their precise roles in the pathogenesis of this disease remain unelucidated at the moment [1, 11].

### Diagnosis, Pathophysiology and Symptoms of Polycythemia Vera, Essential Thrombocythemia and Primary Myelofibrosis

To establish a diagnosis of PV, the 2008 WHO diagnostic criteria include the presence of erythrocytosis and the *JAK2*^V617F ^or *JAK2* exon 12 mutation (major criteria), as well as the presence of one minor criterion, such as bone marrow biopsy consistent with PV (hypercellularity and panmyelosis with prominent trilineage proliferation), low serum erythropoietin (EPO) level, or endogenous erythroid colony (EEC) formation *in vitro*. In the *absence* of *JAK2* mutation, a PV diagnosis is established if two of the aforementioned minor criteria are reached. Diagnosis of ET requires sustained *unexplained* (i.e., non-secondary) thrombocytosis to any value greater than normal (>450 × 10^9^/L), the presence of *JAK2*^V617F^ or another clonal marker, and a bone marrow specimen showing proliferation of atypical megakaryocytes without an increase or left shift in granulopoiesis and erythropoiesis. Criteria for PMF include the presence of bone marrow histologic changes (megakaryocyte proliferation and atypia accompanied by either reticulin and/or collagen fibrosis or increased bone marrow cellularity featuring granulocytic proliferation and decreased erythropoiesis); the absence of an alternative myeloid neoplasm (e.g., CML); and *either* a clonal marker *or* no evidence of the marrow fibrosis being related to an inflammatory or reactive state; as well as two out of four minor criteria; the latter comprise anemia, leukoerythroblastosis, increased serum lactate dehydrogenase levels, or splenomegaly [2, 3].

The cardinal features of the *BCR-ABL1*-negative MPN are the presence of an increased red-cell mass in PV, an increase in the platelet count in ET, and bone marrow fibrosis in PMF [13, 14]. Extramedullary hematopoiesis is often present in MF and may result in splenomegaly and hepatomegaly. Median life expectancy in PV patients can exceed 15–20 years, although the emergence of PPV-MF can significantly shorten survival to a median survival of 5.7 years [15]. A dynamic scoring system based on hemoglobin level <10 g/dL, platelet count <100 × 10^9^/L, and white blood cell (WBC) count >30 × 10^9^/L has been developed to predict survival at any time from diagnosis of PPV-MF [16].

Symptoms of PV commonly include headaches, fatigue, dizziness, pruritus, excessive sweating, and erythromelalgia. Epistaxis, gingival bleeding, and gastrointestinal (GI) bleeding are caused by the increased red blood cell (RBC) mass or acquired von Willebrand disease (vWD), the latter explained by the fact that substantial increases in platelet numbers deplete the large von Willebrand factor multimers that are required to maintain normal hemostasis [17]. Abdominal pain and early satiety due to splenic encroachment are caused by splenomegaly [13, 14]. 

Risk stratification in PV estimates the likelihood of thrombotic complication. High risk is classified as age >60 years and a previous history of thrombosis. The association between platelet counts and risk of bleeding is unclear, but extreme thrombocytosis (platelet count >1,500 × 10^9^/L) may be associated with acquired vWD and bleeding tendency [18–20]. Shortened survival in PV patients is associated with age >60 years, leukocytosis, history of thrombosis, and anemia [20]. 

There are currently no FDA-approved treatments for PV, although several guidelines, such as the European LeukemiaNet (ELN) have reflected efforts to guide the clinician in the appropriate use of currently available treatment modalities (including pharmacotherapy) [19–21]. The goals of therapy are to avoid or manage thrombohemorrhagic events and control systemic symptoms. ‘Conventional’ therapy often involves the use of phlebotomy and low-dose aspirin as first-line therapy for low-risk patients [19–21]. Phlebotomy serves to reduce the RBC mass and blood viscosity, improve platelet function, increase plasma volume, restore systemic and pulmonary pressures, and decrease the risk of thrombotic events. In most patients, there is a concurrent reduction in the symptoms associated with hyperviscosity [22, 23]. Phlebotomy can also sometimes provide relief from pruritus, although some patients require H1 and H2 blockers, ataractics, hydroxyurea (HU; hydroxycarbamide), interferon alpha (IFN)-α, or psoralen with ultraviolet light (A and B) exposure [24, 25]. Aspirin can relieve microvascular complications of erythromelalgia and ocular migraine in PV, but must be used with caution when the platelet count is in excess of 1,000,000/μL because it can cause serious bleeding in cases of acquired vWD [20]. The efficacy of low-dose aspirin (81 mg daily) for preventing thrombotic complications without increasing the risk of major bleeding in PV has not been proven [26]. However, the European Collaboration on Low-Dose Aspirin in Polycythemia Vera (ECLAP) trial in 518 PV patients favored a risk reduction with aspirin in terms of nonfatal myocardial infarction, nonfatal stroke, or death from cardiovascular causes, and the incidence of major bleeding episodes was not significantly increased in the aspirin group as compared with placebo (relative risk, 1.62; 95% confidence interval [CI], 0.27–9.71) [27].

Therapy with HU or IFN-α may be considered in PV patients with splenomegaly and hepatomegaly and in high-risk patients. HU may be effective in reducing thrombotic complications that are not the major cause of death in PV patients. However, HU may play a role in increasing the rate of leukemic transformation, although this is not definitely established; leukemic transformation is a cause of death in PV patients [28–31]. A large randomized trial conducted by the Myeloproliferative Disorders Research Consortium is currently being performed throughout the United States and Europe comparing HU and pegylated (PEG)-IFN-α-2-a in the treatment of patients with high-risk PV. This trial will provide an opportunity to evaluate the leukemogenic risk of HU in this setting. 

Therefore, IFN-α may be the treatment of choice for PV patients younger than 60 years with no history of severe depression, autoimmune disease, and peripheral neuropathy [32]. The use of recombinant IFN-α (rIFN-α-2b) or PEG-IFN-α-2a in the treatment of PV can result in a significant reduction in the phlebotomy rate and is effective in inducing hematologic remission and in some cases reducing expression of *JAK2*^V617F^ [33]. A phase II multicentre study of PEG-IFN-α-2 in 37 evaluable patients resulted in a hematologic response in all patients, including 94.6% complete responses (CR). After the first year, 35 patients remained in hematologic CR, and median %*V617F* decreased from 45% before pegylated IFN-α-2a to 22.5, 17.5, 5, and 3% after 12, 18, 24, and 36 months, respectively. Undetectable levels of *JAK2*^V617F^ was achieved in 7 patients for 6 to 18 months and persisted in 5 patients after treatment discontinuation. No vascular events were associated with treatment [33]. In addition, low doses of rIFN-α have resulted in a decrease in spleen size and successful treatment of the hypercellular phase of PMF and fibrosis that occur after PV [32]. 

ET is characterized by isolated thrombocytosis without obvious cause and proliferation of atypical megakaryocytes in the bone marrow [34]. Symptoms include erythromelalgia, hemorrhage, transient ischemic attacks (including reversible ischemic neurologic defects), microvascular ischemia of the digits, as well as a variety of constitutional symptoms, including headache, visual disturbances, chronic fatigue, and pruritus. Complications include large-vessel arterial or venous thrombosis, especially in patients >60 years of age, those with history of prior thrombosis, and cardiovascular abnormalities (including anatomic heart and vessel abnormalities, e.g., patent foramen ovale). The above notwithstanding, ET patients on aggregate have a relatively preserved life expectancy [35–37].

‘Conventional’ treatment options for ET are limited. HU is often the therapy of choice for high-risk ET patients (age >60 years, with a previous history of thrombosis, cardiovascular risk factors or major hemorrhage, platelet count >1,500 × 103/μL) [38], although there is concern about HU’s leukemogenicity with long-term treatment; other therapies include IFN-α or anagrelide [39]. IFN-α therapy shows clinical efficacy in controlling myeloproliferation and relieving pruritus and other constitutional symptoms in ET: PEG-IFN-α-2a at 90 µg weekly resulted in an overall hematologic response rate of 81% in 39 ET patients and a decrease in *JAK2*^V617F^ allele burden [32, 40]. However, anagrelide was found to be inferior to HU therapy: anagrelide plus low-dose aspirin was associated with increased rates of arterial thrombosis, serious hemorrhage, and transformation to MF compared to HU plus low-dose aspirin in 809 ET patients at high risk for vascular events [41]. In some patients, microvascular symptoms can be managed with low-dose aspirin (81 mg/day) alone or with the addition of clopidogrel [42, 43].

PMF, and the phenotypically related PPV-MF and PET-MF are associated with bone marrow fibrosis, anemia, often progressive splenomegaly, hepatomegaly, and various debilitating symptoms, including the constitutional symptoms of fever, weight loss, and night sweats. MF is associated with worsening cytopenias with eventual bone marrow failure and transformation into a myeloid blast phase, which is most often of myeloid phenotype, and mirrors poor-risk acute myeloid leukemia (AML), termed secondary AML or MF in blast phase (MF-BP). Portal or pulmonary hypertension can also develop and cause significant morbidity. MF is associated with a shortened survival, with a median life expectancy of approximately 5 years in aggregate [44, 45]. Patients with MF can be risk stratified by the International Prognostic Scoring System (IPSS by the International Working Group for Myelofibrosis Research and Treatment [IWG-MRT]) based on clinical variables that hold prognostic significance in multivariate analysis [44]. This prognostic scoring system is based on 5 independent clinical factors (age >60, hemoglobin <10 g/dL, peripheral blood blast count ≥1%, presence of constitutional symptoms, and leukocyte count ≥25 × 10^9^/L), all of which have been determined to be predictive of a poor prognosis. Four distinct risk groups can be identified based on the presence of 0 (low risk), 1 (intermediate risk-1), 2 (intermediate risk-2) or ≥3 (high risk) of these variables, with median survivals of 135, 95, 48, and 27 months, respectively (*P* <0.001). The IPSS was developed for prognosis at the time of PMF diagnosis. Subsequently, the Dynamic IPSS (DIPSS) was developed to assess prognosis at any time during the course of PMF. DIPSS uses the same clinical factors as IPSS; however, DIPSS confers a higher prognostic power to anemia [16]. The DIPSS was further refined to the DIPSS-Plus system, which incorporates additional risk factors: transfusion need, thrombocytopenia, and unfavorable karyotype [46]. The potential benefit of a given therapeutic intervention is weighed against its potential toxicity and considered in the context of the individual’s prognosis. This becomes particularly important when considering experimental therapy for a particular patient and the benefit/risk ratio of therapeutic intervention with the attendant risk of the therapy, which in some cases may not be completely known. 

Until November 2011, there were no FDA-approved drug therapies for MF, and most patients received ‘conventional’ treatment when they were substantially symptomatic, in particular as a consequence of worsening cytopenias and splenomegaly. Allogeneic hematopoietic cell transplantation (allo-HCT) remains the only treatment approach which can offer a possible cure, but it is associated with not insignificant risk of morbidity and mortality [47, 48]. Thus, the risk of allo-HCT in low- or intermediate-risk patients and older patients who typically suffer from multiple comorbidities may not be justified. Indeed, the 1-year treatment-related mortality and overall survival (OS) associated with conventional-intensity conditioning allo-HCT are estimated at 30% and 50%, respectively. In addition, 3-year disease-free survival, OS, and treatment-related mortality have not been shown to be favorably affected by reduced-intensity conditioning (RIC) in a retrospective review of the database from the Center for International Bone & Marrow Transplant Research (CIBMTR) [49, 50]. A phase II study of RIC allo-HCT in patients with advanced MF was conducted by the Myeloproliferative Disorders Research Consortium (MPD-RC), and after a 24-month median follow-up for survivors, 78% of patients in the related group versus 44% in the unrelated group at 12-month follow-up were alive. This study suggests that allo-HCT with RIC and an HLA-matched related donor remains a reasonable therapeutic approach in select MF patients with advanced disease and anticipated significantly abbreviated survival. Studies such as these indicate the need for a risk-benefit assessment of the use of allo-HCT versus ‘conventional’ or investigational pharmacotherapy [47–49,^ 51^]. 

‘Conventional’ drug therapies including corticosteroids, androgen preparations, erythropoiesis stimulating agents (ESA), androgens (danazol), immunomodulators (thalidomide/lenalidomide), splenectomy, splenic irradiation, INF, and cytoreductive therapy (such as HU, busulfan, and melphalan) have been used in patients with MF to address leukocytosis or thrombocytosis, marked splenomegaly, and constitutional symptoms [43, 52]. None of these modalities have ever been shown to consistently, reliably, and durably impact the clinical manifestations (symptoms and signs) of this malignancy in the setting of large, randomized controlled clinical trials. Only one of these agents, notably interferon, has been capable of at least partially reversing or forestalling bone marrow fibrosis, and in select patients restoring hematologic indices [32, 53]. Most importantly, none of these mentioned therapies has ever proven to improve survival.

Following the seminal discovery of the JAK2 mutation and the role of dysregulated JAK-STAT signaling in MPN, there have been many efforts to improve the treatment of patients with MPN, in particular MF [11, 54]. Currently there are several drug classes, including JAK2 inhibitors, pegylated IFN, and immunomodulatory agents in various phases of clinical trials in patients with MF. In this review we summarize the scenario pertaining to the JAK inhibitors, with a detailed medicinal chemistry description of the FDA-approved JAK1 and JAK2 inhibitor, ruxolitinib, indicated for patients with intermediate or high-risk MF (either primary, PPV-MF, or PET-MF).

## JAK SIGNALING AND THE MYELOPROLIFERATIVE DISORDERS

### Overview of JAK Signaling

JAKs are a family of non-receptor tyrosine kinases (NRTKs). The genes coding for JAKs were cloned by polymerization-chain reaction and low-stringency hybridization during searches for novel protein TKs. JAK1 and 2 were initially known as ‘Just Another Kinase’, but were renamed Janus kinase to reflect the highly homologous kinase domains, one of which was later shown to in fact be a pseudokinase [55, 56].

JAKs are critically involved in cell growth, survival, development, and differentiation of hematopoietic and immune cells. They provide the principal signaling pathway for a variety of hematopoietic cytokines and growth factors that depend on signal transmission by cytoplasmic NRTKs [57]. There are four JAK family members in mammals, JAK1, JAK2, JAK3, and TYK2. In humans, the JAK1 gene is located on chromosome 1p31.3, JAK2 is on 9p24, and JAK3 is clustered at 19p13.1 with TYK2 at 19p13.2 [58, 59]. JAKs are relatively large proteins of more than 1,100 amino acids and molecular masses of 120–140 kDa. There are seven JAK homology domains (JH1–JH7) shown in Fig. (**1**). The JH1 domain is a catalytically active TK located at the carboxyl terminus. JH1 is immediately adjacent to JH2, an auto-inhibitory pseudokinase domain. At the amino terminus there is an SH2-like domain (JH3–JH4) and a ‘F-Band-4.1’, ezrin, radixin, moesin (FERM)-like domain (JH6–JH7) [60]. The role of the SH2 domain may involve scaffolding rather than signaling, as a mutation of the SH2 domain in JAK1 did not affect its kinase activity or receptor binding, although SH2 has a key role in other kinases [61]. The FERM domain may mediate interactions with transmembrane proteins and regulate catalytic activity [54].

In mammals, JAK1, JAK2, and TYK2 are ubiquitously expressed, while JAK3 is restricted to hematopoietic cells and immune cells. JAK1 and JAK2 are involved in IFN-γ signaling and physically associate with receptors for type II cytokines such as interleukin (IL)-6, IL-10, IL-11, IL-19, IL-20, and IL-22; JAK2 is activated by hormone-like cytokines such as growth hormone, prolactin, erythropoietin (EPO), thrombopoietin (TPO), as well as those involved in hematopoietic cell development including IL-3 and granulocyte macrophage colony-stimulating factor; JAK1 and JAK3 associate with γc cytokines, including IL-2, IL-4, IL-7, IL-9, IL-15, and IL-21; and, finally, TYK2 associates with cytokine receptors that signal through various combinations with JAK1 and JAK2, such as type 1 IFNs and the p40-containing cytokines IL-12 and IL-23 [54].

At the cellular level, JAKs localize from the cytoplasm close to the plasma membrane and specifically bind (in a non-covalent fashion) to the intracytoplasmic domain of various cytokine/growth factor receptors by their FERM-like domain. Ligand binding to the cognate cytokine or growth factor ‘kinase-less’ receptor promotes a conformational change in the receptor and multimerization of receptor subunits which brings JAKs into close proximity; this subsequently allows trans-phosphorylation and activation. Activated JAKs in turn phosphorylate specific tyrosine residues on the cytokine receptors, thus promoting recruitment and phosphorylation of downstream signaling molecules, including STATs (signal transducers and activators of transcription), phosphatidyl-inositol-3-kinase (PI3K), protein kinase-B (also known as AKT), and the mitogen-activated protein kinases (MAPKs). STATs comprise the main family responsible for mediating signals derived from cytokine receptors and assorted growth factor receptors via JAK activation. STATs bound to cytokine receptors are phosphorylated by JAKs at a conserved tyrosine residue near the C-terminus. This phosphotyrosine interacts with the conserved H2 domain of STATs to cause STAT dimerization. Subsequently, phosphorylated dimerized STATs enter the nucleus by an importin α-5 or Ran nuclear import-dependent pathway and bind specific regulatory sequences in select gene promoter regions to activate or repress transcription of target genes, thus leading to eventual corresponding biologic and physiologic effects [63].

A number of effector proteins that facilitate JAK activation have been identified. These include STAMs (signal-transducing adapter molecules), StIPs (STAT-interacting proteins), and select members of the SH2B/Lnk/APS family. STAMs can facilitate the transcription of target genes [64]; StIPs increase the phosphorylation of STATs by JAKs [65]; and SH2-Bβ increases JAK tyrosine phosphorylation and catalytic activity [66]. There are three major classes of negative regulators of the JAK/STAT pathway: SOCS (suppressors of cytokine signaling), PIAS (protein inhibitors of activated STATs), and PTPs (protein tyrosine phosphatases). The SOCS may bind phosphotyrosines on receptors and prevent them from activating downstream signaling molecules; they may bind directly to JAKs or JAK-associated cytokine receptors and inhibit JAK kinase activity; or they may decrease the stability of the said receptors as well as JAKs through ubiquitination [65, 67]. The PIAS proteins bind to phosphorylated STAT dimers and prevent them from interacting with target genes, while PTPs dephosphorylate JAKs and receptors [65].

### JAK Signaling in MPN

In 2005, a major advance in the understanding of the pathogenesis of MPN occurred when *JAK2*^V617F^, a clonal recurrent point mutation in the pseudokinase domain of JAK2, was identified in many patients with MPN [4–8]. *In vivo* studies showed that expression of *JAK2*^V617F ^in murine bone marrow transplant models resulted in a PV phenotype with erythrocytosis, leukocytosis, and splenomegaly, a clinicohematologic phenotype that progressed to MF in the mouse in about 3 months. Thrombocytosis was not observed, thus suggesting the involvement of additional genetic events influencing the phenotype of ET and MF (which are both inextricably associated with megakaryocytic proliferation and atypia) [5, 54].

*JAK2*^V617F^ is a guanine to thymidine point mutation that results in a valine (Val; V) to phenylalanine (Phe; F) substitution at codon 617 within the JH2 domain of *JAK2*. The corresponding protein is a constitutively active TK that is phosphorylated at the activation loop Y1007 and disturbs/partially abrogates the influence of JH2 on the JH1 kinase domain; this influence is normally auto-inhibitory to the JH1 catalytic domain. In this sequence of molecular events, the *JAK2*^V617F^ mutation results in a ‘gain of function’ (GOF) modification that confers cytokine hypersensitivity and cytokine-independent growth to hematopoietic cells [4–8]. In addition, the *JAK2*^V617F^ mutation may increase the stability of the JAK2 protein by rendering it resistant to the effect of SOCS3 binding, as overexpression of SOCS3 results in increased SOCS3 and *JAK2*^V617F^ phosphorylation [68].

The *JAK2*^V617F^ mutation may occur in a hemangioblast, a stem cell common to a hematopoietic stem cell and an endothelial cell [69]. It is likely that clonal expansion occurs at later stages of differentiation, as *JAK2*^V617F^ allele burden increases in hematopoietic stem cells and progenitors as disease progresses [54]. Of note, the situation is in reality more complex, as the exact phenotype of the disease resulting from the presence of the *JAK2*^V617F^ mutation may be dependent on gene dosage, which directs the level of signaling; in transgenic mouse models, the level of *JAK2*^V617F^ expression seems to determine whether the disease phenotype resembles PV or ET [54, 70]. 

A cytokine receptor scaffold may be required for *JAK2*^V617F^-mediated hematopoietic cell transformation as *JAK2*^V617F^-mediated transformation to cytokine-independent growth appears to be most efficient in cells that coexpress the EPO, TPO, and granulocyte colony-stimulating factor homodimeric type I cytokine receptors. These data may also explain the multi-lineage expansion seen in *JAK2*^V617F^-positive MPN [71]. Furthermore, *JAK2*^V617F^ activation appears to involve a mechanism that requires the FERM domain, as constitutive activation of *JAK2*^V617F^ is prevented by the Y114A mutation in the FERM domain, and introduction of the *V617F* mutation in a protein composed only of the JH1–JH2 domains does not result in constitutive signaling [54]. 

*JAK2*^V617F^-mediated cellular transformation seems to involve activation of JAK2-STAT3, JAK2-STAT5, ERK1/2 MAPK, and PI3K/AKT downstream signal transduction pathways, as: (i) STAT3 and BCL_X_ overexpression are characteristic findings in human PV, (ii) human hematopoietic progenitors expressing constitutively active STAT5 and its target BCL_X_ undergo EPO-independent colony formation, and (iii) hematopoietic transformation in murine bone marrow by TEL-JAK2 requires STAT5 [52]. *JAK2*^V617F^ can also induce its effects directly, as it can translocate to the nucleus of leukemic cells and primary CD34+ hematopoietic progenitors to phosphorylate histone H3 at tyrosine 41 (H3Y41), decrease the affinity of histone H3 for the transcriptional repressor heterochromatin protein 1a (HP1a), and promote the expression of genes involved in cell proliferation and survival [72]. The molecular basis for the involvement of the JAK/STAT pathway in neoplastic transformation in MPN (reflecting a torrent of recent data) is summarized in Fig. (**2**).

The *JAK2*^V617F^ mutation is present in more than 95% cases of PV, and over 50% cases of ET and PMF [9]. Recently, research has been directed at understanding MPN cases that *lack* the *JAK2*^V617F^ mutation. In *JAK2*^V617F^-negative PV patients, GOF mutations in exon 12 of JAK2 that confer growth factor independence in Ba/F3-EPO-R cells have been described [73]. Four of these occur in the region between SH2 and JH2 and involve a point mutation, a double mutation, a 2-amino acid deletion, and a 2-amino acid deletion followed by an insertion in residues 537–543. *JAK2*^V617F^-negative PV patients with mutations in exon 12 are phenotypically distinct from those that are *JAK2*^V617F^-positive, as they present with isolated erythrocytosis instead of a trilineage expansion. The reasons for this remain unclear, but the exon 12 mutant protein may exhibit a higher constitutive kinase activity or increased affinity for the EPO receptor (EPO-R). Mutations in exon 12 have not been reported in ET or PMF [73].

### The Genetic Complexity of MPN

Although *JAK2*^V617F^ is a dominant mutation in MPN, many others have been described at a lower frequency [1, 74]. These include mutations in the cytokine/growth factor receptors that bind JAK2, such as the TPO receptor (TPO-R; *MPL*)*.* *MPL* mutations are present in 5–10% PMF patients and 2–5% of those with ET. They (*MPL*^W515L/K/N/A^) occur in a stretch of five amino acids (K/RWQFP) adjacent to the transmembrane domain of this receptor. These five amino acids prevent spontaneous activation of the receptor. *MPL*^W515 ^mutations activate the TPO-R and are characterized by spontaneous megakaryocyte growth in the absence of EEC formation. A murine *MPL*^W515L^ expression model results in a JAK2-dependent lethal MPN in the mouse characterized by thrombocytosis and MF [75]. Other genetic underpinnings of importance are alterations that affect epigenetic regulation of transcription, including mutations in *TET2 *and* DNMT3A* as well as the recent discovery of mutations and copy-number loss in multiple members of the Polycomb Repressive Complex 2 (PRC2) [76–81].

*CBL *(casitas B-lineage lymphoma) gene mutations have been found in <10% PMF cases [82]. The *CBL *gene family codes for proteins with E3-ubiquitin ligase activity. The c-CBL and CBL-B proteins are involved in the ubiquitination of growth factor receptors and negatively regulate signal transduction pathways activated by tyrosine kinases. *c-CBL* and *CBL-B* mutations have been found in cases of post-MPN-AML. In the absence of wild-type functional *CBL*, *c-CBL* mutants cause a GOF effect and hypersensitivity to various cytokines including SCF, TPO, FLT3 ligand, and IL-3. However, the functional significance of these findings in the pathogenesis of MPN is unknown [83, 84].

The search for pre-JAK2 molecular events in MPN led to the discovery of *TET2* (ten eleven translocation-2) and *ASXL1* (additional sex comb like-1) defects [71]. *TET2* belongs to a family of three genes and may participate in a DNA demethylation process. Multiple mutations of the *TET2* gene have been identified in 12% MPN, 14% PV, 8% ET, 20% PMF, and 25% post-MPN-AML patients [71, 85, 86]. Most *TET2* mutations encode truncated proteins as a result of nonsense mutations or small insertions or deletions leading to a frame shift. Some patients have missense mutations localized in the two highly conserved domains, missense mutations located outside the conserved domains, or splicing site mutations. MPN *TET2* mutations usually involve one copy of the gene; however, in a minority of patients, two defects are found. The role of *TET2* in the pathogenesis of MPN is still being deciphered, but may involve modulation of epigenetic mechanisms that lead to dysregulation of expression of genes involved in early hematopoiesis and myeloid differentiation. Mutations involving the loss of two functional copies of the *TET2* gene may participate in the initiation of a premalignant clone and the progression of the disease [71].

*ASXL1* belongs to a family of three genes that are involved in the control of development-related genes through chromatin remodeling. Mutations in *ASXL1* are located in exon 12 and result in a truncated protein. They are found in 15% cases MPN during the chronic phase of the disease and in 20% cases of post-MPN-AML [71, 84].

Other mutations occur in genes which include: (i) *LNK* which encodes a plasma-membrane adaptor protein responsible for inhibiting wild-type and mutant JAK2 signaling: loss of function mutations in *LNK* have been identified in ET and PMF patients; (ii) enhancer of zest homologue 2 (*EZH2*) which is involved in epigenetic repression of apoptosis and stem cell renewal: mutations are found in 13% MF patients; (iii) *NF1* (neurofibromatosis-1) which is a negative regulator of the RAS signal transduction pathway: loss of *NF1* can lead to progressive MPN; (iv) *IDH1/2* mutations which result in the production of 2-hydroxyglutarate; these are found in 5% ET and PMF patients and 21% post-MPN AML cases; and (v) *IKZF* which encodes the transcription factor Ikaros (zinc finger-containing protein) and has a role in the regulation of hematopoiesis: mutations found in 0.2% MPN [1, 71]. The role of these varied mutations in the pathogenesis of MPN is unknown and likely complex, but may be important in the modulation of signaling pathways and the epigenome contributing to progression of disease and leukemic transformation [11]. This information is summarized in Fig. (**3**).

Further genetic complexity of MPN occurs at the microRNA (miRs) level. miRs are non-coding 18–22 nucleotide RNAs that regulate gene expression by destabilizing specific target mRNA or inhibiting protein translation. miRs are important regulators of hematopoiesis and may act as tumor suppressors (e.g., miR-15/16) or oncogenes (e.g., the miR-17–92 cluster) in the pathogenesis of some acquired hematologic disorders. Some hematopoietic cell lineages and hematologic diseases have defined miR signatures, including abnormally high expression of miR-155 in Hodgkin lymphoma and diffuse large B-cell lymphoma, loss of expression of miR-15 and miR-16 in chronic lymphocytic leukemia, and aberrant expression of let-7a, miR-182, miR-143, miR-145, miR-223, miR-26b, miR-30b, miR-30c, and miR-150 in granulocytes, mononuclear cells, platelets, or reticulocytes of PV patients (with correlations between aberrant expression of miR-143, let-7a, miR-30c, miR-342, and miR-150 and *JAK2*^V617F ^mutation frequency). These data provide further insight into the complex molecular pathogenesis of MPN [87].

### The Role of JAK2 and JAK1 Inhibitors in MPN

Discovery of the different molecular pathways critical for development of MPN have enabled the identification of more specific diagnostic criteria and the rational design and development of targeted therapies including JAK inhibitors, such as ruxolitinib (INCB018424), TG101348 (SAR 30253), lestaurtinib (CEP701), CYT387, pacritinib (SB1518), AZD1480, XL019, LY2784544; the mTOR inhibitor everolimus; the epidermal growth factor receptor (EGFR) inhibitor erlotinib; the proteasome inhibitor (that affects the NF-êB pathway) bortezomib; as well as the histone deacetylase inhibitors givinostat (ITF2357), panobinostat (LBH589), and vorinostat that are epigenetic agents affecting chromatin remodeling pathways [88].

Therapies that inhibit JAK1 and JAK2 catalytic activities have been designed and developed in response to the discovery of dysregulated JAK-STAT signaling in MPN patients, regardless of the source(s) of this dysregulated activity. Clinical data on selective inhibitors of JAK2 or JAK1 and JAK2 are promising and suggest that they improve certain MPN-related symptoms and constitutional signs, as well as reduce splenomegaly. As yet, it has not been shown that these agents can reverse or forestall the hematopathologic features of MPN or modulate disease progression at a cellular/molecular level. The clinical efficacy of JAK inhibitors has been attributed to a general dampening of cytokine signaling, a fact which is particularly pertinent for JAK1 and JAK2 inhibitors. Class I JAK2 inhibitors act by competing for ATP-binding in the catalytic site and are not specific for the *JAK2*^V617F ^mutation in the distant pseudokinase domain. As a result, these agents inhibit both mutant and wild-type JAK2. Inhibition of wild-type JAK2 partially blocks intracellular signaling via the EPO-R and TPO-R required for normal hematopoiesis, thus resulting in predictable (expected) and dose-dependent anemia and thrombocytopenia. Class II JAK2 inhibitors affect constitutive homeostasis through their indiscriminate inhibition of non-JAK2 TKs and may be associated with various toxicity profiles [88, 89].

Several JAK inhibitors are presently being evaluated in various stages of clinical development [89]. Many of these agents have clinical trial data available, with conclusions from randomized controlled multicenter trials providing a higher level of evidence than results from small single-arm studies (Table **3**): (i) TG101348 exhibits selectivity for JAK2 and *JAK2*^V617F ^compared with other JAK kinases. Phase I clinical data of TG101348 in 59 PMF, PET-MF, or PPV-MF patients showed a significant decrease in allele burden at 6 months in mutation-positive patients (n = 51; *P* = 0.04), improved constitutional symptoms such as early satiety, night sweats, fatigue, pruritus, and cough, a modest reduction in serum cytokine levels, a spleen response per IWG criteria by six and 12 cycles of treatment in 39 and 47% of patients, respectively, and normalization of blood counts in the majority of patients with leukocytosis or thrombocytosis at baseline after six (57 and 90%, respectively) and 12 (56 and 88%, respectively) cycles [90]; (ii) Lestaurtinib is an oral multikinase inhibitor. It is active against both wild-type JAK2 and *JAK2*^V617F^as well as FLT3. Phase I/II clinical data of lestaurtinib showed little reduction in *JAK2*^V617F^ allele burden but a median reduction of 6.4 cm in spleen size in 37% of 19 evaluable *JAK2*^V617F^-positive PMF, PET-MF, or PPV-MF patients [91, 92]; (iii) CYT387 is a JAK1 and JAK2 inhibitor. Phase I/II clinical data of CYT387 in 60 patients with MF (68% PMF) suggested clinical improvements in anemia and splenomegaly in 50% of evaluable patients and 47% of patients with baseline splenomegaly, respectively. Treatment was not associated with a decrease in *JAK2*^V617F ^allele burden [93–95]; (iv) pacritinib is a JAK1, JAK2, and TYK2 inhibitor. Phase II clinical data of 400 mg pacritinib in 34 MF patients showed a >50% reduction in palpable spleen volume in 44% of patients and significant improvement in MF-related symptoms (abdominal pain, bone pain, early satiety, inactivity, night sweats, pruritus) at 6 months compared to baseline. A phase III clinical study for pacritinib in MF patients is planned [96]; and (v) AZD1480 exhibits marked JAK2 selectivity. AZD1480 is currently in an ongoing phase I clinical trial [97]. 

## THE CHEMICAL AND PHARMACOLOGICAL PROPERTIES OF RUXOLITINIB

### Preclinical Characterization of Ruxolitinib

Ruxolitinib (*R*)-3-(4-(7*H*-pyrrolo2,3-*d*pyrimidin-4-yl)-1*H*pyrazol-1-yl)-3-cyclopentylpropanenitrile phosphate, formerly known as INCB018424 or INC 424, is a rationally designed potent, and orally bioavailable inhibitor of JAK1 and JAK2. It has a molecular formula of C_17_H_21_N_6_O_4_P and a molecular weight of 404.36. *In vitro* enzyme assays confirm that ruxolitinib inhibits JAK1 and JAK2 with IC_50_ values of 3.3 nM and 2.8 nM, respectively; IC_50_ values are approximately 6-fold greater for TYK2 and ≥130-fold greater for JAK3 [98].

Studies were conducted in cytokine-dependent Ba/F3 cells transformed to growth factor independence by ectopic expression of *JAK2*^V617F^ and a requisite type I cytokine receptor (EPO). Nanomolar concentrations of ruxolitinib inhibited constitutive *JAK2*^V617F^, STAT5, and ERK1/2 phosphorylation, resulting in reduced cellular proliferation with an IC_50_ = 127 nM, and promoted induction of apoptosis. Ruxolitinib treatment of Ba/F3 cells transformed with the BCR-ABL-1 oncoprotein and activating mutations in c-KIT had no effect on cell viability or BCR-ABL-1 signaling, confirming the selective nature of ruxolitinib inhibition. In primary culture of mononuclear cells expressing the *JAK2*^V617F ^mutant allele at frequencies >90% from patients with PV, ruxolitinib treatment resulted in dose-dependent inhibition of erythroid (IC_50_ = 223 nM) and myeloid (IC_50_ = 444 nM) progenitors and EEC formation (IC_50_ = 67 nM). *In vivo*, the efficacy of ruxolitinib in a murine *JAK2*^V617F^-driven malignancy model was demonstrated by the fact that it significantly reduced splenomegaly, decreased elevated levels of circulating IL-6 and tumor necrosis factor (TNF)-α to normal, and increased survival, without causing anemia or lymphopenia [98].

### Metabolism, Excretion, Pharmacokinetics and Pharmacodynamics of Ruxolitinib

Clinical metabolism and pharmacokinetic (PK) studies of oral doses of [^14^C]-ruxolitinib were conducted in healthy adults. Ruxolitinib and associated radioactivity were rapidly absorbed into blood cells and plasma, with peak plasma concentrations (C_max_) attained within 1 hour (T_max_) which declined in a monophasic (t_1/2_ = 2.32 h) and biphasic manner (t_1/2_ = 5.81 h). Oral doses were >99% metabolized with <1% of the dose recovered as parent drug. Recovery of administered radioactivity was rapid (>70% within 24 h post dose); a total of 96% was recovered (74% urine, 22% feces), indicating that ruxolitinib was >95% absorbed. After oral dosing, 97% of radioactivity in the circulation could be accounted for by parent drug and identified metabolites. Parent drug represented 58–74% total radioactivity in the circulation up to 6 hours post dose. A total of eight metabolites were identified with two major metabolite peaks (M18 and a peak containing M16/M27, both hydroxylations on the cyclopentyl moiety of the parent molecule). Other circulating peaks included mono- and dihydroxylated metabolites. Half-lives ofradioactivity showed that the clearance of metabolites was slightly extended compared to the parent drug but individual metabolites were undetectable 24 hours post dose. Furthermore, in healthy subjects receiving daily oral doses of unlabeled ruxolitinib, there were minimal differences in parent and metabolite concentrations between day 1 and day 10. Taken together, these data suggest that accumulation of metabolites during multiple dosing regimens is unlikely [99].

Pharmacokinetic and pharmacodynamic (PK/PD) studies were performed to investigate the effects of polypharmacy on the dosing paradigm for ruxolitinib. Ruxolitinib is primarily metabolized by cytochrome P450 enzyme, CYP3A4; therefore, the potential effects of coadministering the CYP3A4 inhibitors ketoconazole and erythromycin and the CYP3A4 inducer rifampin with ruxolitinib were evaluated in healthy adults. Coadministration of ketoconazole (a potent CYP3A4 inhibitor) decreased oral clearance by 48% and increased total ruxolitinib plasma exposure by 91%. Concomitant treatment with ketoconazole also resulted in a 2-fold increase in ruxolitinib-induced inhibition of IL-6 stimulated STAT3 phosphorylation in whole blood (the area under the pharmacodynamic effect curve [PD AUCE_0-sub>0-t_] of IL-6 stimulated STAT3 phosphorylation, a PD index for the drug); the geometric mean ratio for PD AUCE_0-t_ between the ruxolitinib plus ketoconazole coadministration and ruxolitinib alone was 2.0 (90% CI 1.5–2.6; *P* = 0.0004). Coadministration of erythromycin (a weaker CYP3A4 inhibitor) increased plasma ruxolitinib exposure by 27%. Induction of CYP3A4 with rifampin decreased plasma ruxolitinib exposure by 71% and its PD effect by only 10%. This discrepancy between the magnitude of the PK and PD effects with rifampin coadministration may be explained by an increase in the relative abundance of ruxolitinib metabolites (which have pharmacologic activity). Collectively, these data indicate that dosing regimens including ruxolitinib and potent CYP3A4 inhibitors may need to be modified accordingly [100]. 

## RUXOLITINIB CLINICAL TRIAL PROGRAM IN PATIENTS WITH MPN

Ruxolitinib is currently the first JAK inhibitor to have completed phase III trials successfully in patients with MPN. Based on the robust phase III study results of patients with MF and other data from the MF development program, ruxolitinib was approved on November 16, 2011 by the US FDA for the treatment of intermediate or high-risk MF (including PMF, PPV-MF, and PET-MF).

### A Phase I/II Study of the Efficacy and Safety of Ruxolitinib for Patients With Primary Myelofibrosis, Post-Polycythemia Vera-Myelofibrosis, or Post-Essential Thrombocythemia-Myelo-fibrosis

The efficacy and safety of ruxolitinib were investigated in a phase I/II study (INCB018424-251; ClinicalTrials.gov number, NCT00509899) of 153 patients with *JAK2*^V617F^-positive or *JAK2*^V617F^-negative PMF (53%), PET-MF (15.2%), or PPV-MF (31.8%). Patients had a median age of 65 years, 82% were *JAK2*^V617F^-positive, and 92% had palpable splenomegaly with a median palpable spleen length of 19 cm; 65% patients were in the high-risk category according to the IPSS, with an expected median survival of 27 months. In the phase I portion of the trial, the dosing schedule was 25 mg twice daily (BID) with a subsequent increase to 50 mg BID, or once daily doses of 25–200 mg. In the phase II portion of the study, dosing schedules were 25 mg BID with a reduction to 10 mg BID after 2 months; 10 mg BID with dose escalation if there was no response and no toxicity; and 10 mg BID in patients with baseline platelet counts of 100–200 × 10^9^/L or 15 mg BID in patients with baseline platelet counts of >200 × 10^9^/L, with allowance for dose increases up to 25 mg BID if there was no response and no toxicity [101].

In the phase I portion of the study, an MTD of 25 mg BID was established based on the presence of clinically significant thrombocytopenia. Phase II identified 15 mg BID with individualized dose adjustment to be an optimal dose for the initiation of a phase III program (see below). This phase II trial confirmed that ruxolitinib treatment is associated with significant clinical activity and a sustained reduction in splenomegaly and alleviation of MF-related debilitating symptoms. Indeed, 52% of patients receiving the 15-mg BID regimen achieved a rapid objective response of a ≥50% reduction in splenomegaly which was sustained for >12 months; this included a median 33% reduction in spleen volume (as measured by splenic magnetic resonance imaging [MRI] in a subset of patients) and a median 52% decrease in palpable spleen length. After a median treatment duration of 12 weeks, RBC transfusion independence (CI by IWG-MRT) was achieved and maintained for a median duration of 20 months in 14% of patients who were transfusion-dependent at baseline, and mean baseline WBC and platelet counts were reduced and remained stable for 1 year [101].

Subgroup analyses of 34 patients treated with 25 mg of ruxolitinib BID indicated that ruxolitinib significantly improved nutritional status and exercise capacity. Patients on ruxolitinib therapy showed improved appetite and gained weight as the trial progressed (mean increase of 0.40 kg at 1 month, 2.93 kg at 2 months, and 3.70 kg at 3 months). In patients with baseline body mass indices (BMIs) in the lowest quartile, weight gain was more consistent, of greater magnitude, and sustained compared with those in the highest baseline BMI quartile. At enrollment, median total cholesterol of the study population was 95 mg/dL, with levels in 94% and 55% of patients below 150 mg/dL and 100 mg/dL, respectively. Following ruxolitinib treatment, median total cholesterol increased to 145 mg/dL (range, 72–289 mg/dL) with 73% patients increasing their cholesterol above their baseline. In addition, baseline leptin levels in the study population were very low (mean level being 2.55 ng/mL), with leptin levels in ~50% patients below 1 ng/mL (normal range, 6–12 ng/mL). Ruxolitinib treatment increased plasma leptin levels by 176% after 1 month of treatment. After 2 months, plasma leptin increased to levels similar to those observed in healthy volunteers (mean, 7.04 ng/mL; range 0.2–35 ng/mL). These data suggest that ruxolitinib treatment was able to at least partially reverse the markers of chronic malnutrition (which occasionally manifests as frank cachexia) in MF patients [102]. The phase I/II study also showed that ruxolitinib treatment improved exercise capacity as measured by the 6-minute walk test, with increases from baseline of 34, 57, and 71 meters after 1, 3, and 6 months of therapy, respectively [101].

The improvements in clinical data and symptoms in MF patients in this phase I/II study provided clinical proof of concept regarding the ability of ruxolitinib to target the underlying pathophysiology of MF. Additionally, correlative marker results suggest that the mechanism of action of ruxolitinib was at least partially based on decreases in the levels of proinflammatory cytokines, as improvements of clinical symptoms were associated with decreases in the plasma levels of IL-1RA, MIP-1β (macrophage inflammatory protein-1β), TNF-α, and IL-6, which were all elevated at baseline but decreased following ruxolitinib treatment [101]. This information is depicted in Fig. (**4**).

Importantly, in this phase I/II trial, ruxolitinib treatment proved efficacious in patients with both wild-type JAK2 and those with *JAK2*^V617F^ mutation. The proportion of all patients with a response who received 15 mg BID and 25 mg BID was similar among the 61 patients with the *JAK2*^V617F ^mutation and the 11 patients without the mutation (51% vs. 45%). Furthermore, the clinical benefit of ruxolitinib was independent of the magnitude of *JAK2*^V617F^ allele burden. These results suggest that although ruxolitinib does not delete *JAK2*^V617F^-positive clones, it acts to inhibit in a potent and sustained manner overactive JAK2 signaling, as well as JAK1-dependent cascades [101].

The median duration of therapy was >14.7 months, and low-grade nonhematologic toxic effects occurred in <10% of patients. Hematologic adverse events were predictable and included anemia and thrombocytopenia. Of note, thrombocytopenia was less frequent and less pronounced in patients who received the optimal (15 mg p.o. BID) regimen and reversible after dose interruption in those on higher ruxolitinib doses. Serious adverse events (SAEs) that were at least possibly related to treatment occurred in only 12 patients (mainly myelosuppression), and 3 cases of transformation to AML were reported. The overall survival rate was 84% (90% survival when only deaths that occurred during the study were considered) during the follow-up period [101].

Based on the data from this phase I/II study, ruxolitinib was tested in the COMFORT trials, the first controlled, randomized phase III clinical trials in MF.

### COMFORT-I: A Randomized, Double-Blind Phase III Trial of Ruxolitinib Versus Placebo for Patients With Primary Myelofibrosis, Post-Polycythemia Vera-Myelofibrosis, or Post-Essential Thrombocythemia-Myelofibrosis

The clinical benefits of ruxolitinib were demonstrated in COMFORT-I (ClinicalTrials.gov number, NCT00952289), a double-blind placebo-controlled phase III trial of 309 patients aged 40–91 years with intermediate-2 or high-risk (IPSS) PMF, PPV-MF, or PET-MF resistant or refractory to treatment and with palpable splenomegaly (≥5 cm below left costal margin). Patients were randomized to receive placebo (n = 154) or 15 or 20 mg ruxolitinib (n = 155) BID depending on baseline platelet count (100–200 × 10^9^/L or >200 × 10^9^/L, respectively). Crossover from placebo to ruxolitinib was permitted for protocol-defined worsening splenomegaly. The primary endpoint was defined as the proportion of patients with ≥35% reduction in spleen volume at week 24 of therapy, assessed by MRI (or computed tomography [CT]). At baseline, patients had a median spleen volume of >2500 cm^3^ (>10 times normal). Secondary endpoints were durability of spleen response, changes in symptom burden as assessed daily by the modified Myelofibrosis Symptom Assessment Form (MFSAF) v2.0, and overall survival [103]. 

At 24 weeks, 41.9% ruxolitinib-treated patients achieved a ≥35% reduction in spleen volume compared to 0.7% on placebo (*P* <0.001) with a median reduction in spleen volume of 33%. In the placebo group, there was a median increase in spleen volume of 8.5%. In patients treated with ruxolitinib, reductions in spleen volume were apparent by the first measurement at 12 weeks. The mean changes from baseline in individual patients are depicted in Fig. (**5**) [103].

The study confirmed that ruxolitinib treatment is associated with significant improvements in certain debilitating symptoms of MF. The prevalence of MF patients who experienced disease-associated symptoms at baseline was high. In the ruxolitinib-treated group, abdominal discomfort was experienced in 95.3% patients, left subcostal pain in 83.9%, abdominal fullness/early satiety in 94.0%, night sweats in 80.5%, itching in 75.8%, and bone/muscle pain in 81.9%. Additionally, inactivity was documented in 91.9% [104]. Percentages were similar in the placebo group at baseline. At 24 weeks, 45.9% of ruxolitinib-treated patients showed a ≥50% reduction in Total Symptom Score (TSS, which includes scores for abdominal pain, pain under left ribs, early satiety, night sweats, itching, and bone/muscle pain) from baseline compared to 5.3% in the placebo group (*P* <0.001). There was a mean 46.1% improvement in TSS in ruxolitinib-treated patients compared with a mean 41.8% worsening in the placebo group (*P* <0.001). The majority of responders had achieved a response within the first 4 weeks. Each individual symptom of the MFSAF improved in ruxolitinib-treated patients, whereas symptoms worsened in the placebo group (*P* <0.01 for each), as shown in Fig. (**6**). In the ruxolitinib group, 63% and 47% patients, respectively, with a ≥35% and <35% reduction in spleen volume also had a ≥50% improvement in spleen-related symptoms (combined score for abdominal discomfort, pain under left ribs, and early satiety) [103]. 

At 24 and 48 weeks, ruxolitinib-treated patients showed mean reductions in *JAK2*^V617F^ allele burden of 10.9% and 21.5%, respectively; patients receiving placebo had a mean increase of 3.5% and 6.3% [103]. As demonstrated in the phase I/II trial, patients with and without the *JAK2*^V617F ^mutation had clinical benefit from treatment with ruxolitinib.

A survival analysis was conducted at the time of a planned safety update (4 months after the primary analysis data cutoff date). Thirteen patients in the ruxolitinib group and 24 in the placebo group died (median follow-up of 52 and 51 wks, respectively), with a hazard ratio (95% CI) of 0.50 (0.25, 0.98; *P* = 0.04) [103]. 

Ruxolitinib was generally well tolerated in patients with MF. Nonhematologic adverse events (AEs) that were more common in the ruxolitinib versus the placebo group, included ecchymosis (all grades, 18.7% vs. 9.3%), dizziness (all grades, 14.8% vs. 6.6%), and headache (all grades, 14.8% vs. 5.3%); these events were predominantly grade 1or 2. The most common hematologic AEs in the ruxolitinib group were thrombocytopenia and anemia. These were usually managed with dose modifications (transfusions for anemia), and rarely led to discontinuation. Hematology laboratory values are shown in (Table **4**). Patients are included at their worst-on-study grade [103]. 

### COMFORT-II: A Randomized Phase III Study of Ruxolitinib Versus Best Available Therapy for Primary Myelofibrosis, Post-Polycythemia Vera-Myelofibrosis, or Post-Essential Thrombocythemia-Myelofibrosis

The benefits of ruxolitinib compared to best available therapy (BAT; other ‘conventional’ agents or no therapy) were shown in COMFORT-II (ClinicalTrials.gov number, NCT00934544), a phase III clinical trial in 219 patients with intermediate-2 or high-risk (IPSS) PMF, PPV-MF, or PET-MF and palpable splenomegaly (≥5 cm below left costal margin). Patients were randomized (2:1) to receive oral ruxolitinib twice-daily (n = 146) or BAT (n = 73). Therapies in the BAT group included HU, corticosteroids, erythropoiesis-stimulating agents, androgens, IFN, and other agents, with 67% of patients in the BAT group receiving one or more of these therapies. The primary endpoint was defined as the proportion of patients with ≥35% reduction in spleen volume at week 48 of therapy, assessed by MRI (or CT). A secondary endpoint was the proportion of patients achieving a ≥35% reduction in spleen volume at week 24 [105].

For the primary efficacy outcome measure, 28% ruxolitinib-treated patients showed ≥35% reduction in spleen volume compared to 0% in the BAT group (*P* <0.001) at week 48. For the key secondary efficacy outcome measure, at 24 weeks, 32% ruxolitinib-treated patients showed ≥35% reduction in spleen volume compared to 0% in the BAT group (*P* <0.001) [105]. Median time to first ≥35% reduction in spleen volume from baseline was 12.3 weeks in the ruxolitinib arm, and of the 69 patients who achieved ≥35% reduction at any time during the study, 44 (64%) did so at the week 12 assessment. In contrast, only 1 BAT-treated patient achieved a ≥35% reduction in spleen volume by week 12 and this reduction was lost before the week 24 assessment [105]. The prespecified median duration of response in ruxolitinib-treated patients was not reached; however, 80% of patients were still responding at a median 12 months of follow-up [105]. Ruxolitinib treatment was also associated with improvements in quality of life and symptoms associated with MF compared with BAT.

In an analysis based on a planned safety update with approximately 2 months of additional follow-up (median, 61.1 weeks), 11(8%) and 4 (5%) deaths were reported in the ruxolitinib and BAT arms, respectively [105]. The median survival time had not been reached. The authors note that interpretation of these data is limited by significant crossover from BAT to ruxolitinib and limited survival follow-up in some patients. 

Ruxolitinib was generally well tolerated. The most frequently reported nonhematologic AE in ruxolitinib-treated patients was diarrhea (all grades, 23% with ruxolitinib vs. 12% in patients treated with BAT). Peripheral edema was the most frequently reported AE with BAT (all grades, 22% with ruxolitinib vs. 26% for BAT). The most frequently reported grade 3–4 nonhematologic AEs were abdominal pain (3%) with ruxolitinib and dyspnea and pneumonia (4% each) with BAT. Thrombocytopenia and anemia occurred more frequently in ruxolitinib-treated patients vs. BAT-treated patients. Hematology laboratory values are shown in (Table **5**). Patients are included at their worst-on-study grade [105].

### Ruxolitinib Therapy in Patients with Polycythemia Vera and Essential Thrombocythemia Refractory to Hydroxyurea

A single-arm, open-label phase II trial (INCB018424-256; ClinicalTrials.gov number, NCT00726232) investigated the efficacy and safety of ruxolitinib therapy in patients with PV and ET refractory/resistant to (or intolerant of) current treatment regimens [106]. An initial 8-week run-in established 10 and 25 mg BID as starting doses for expansion cohorts of 6–8 PV (n = 34) and ET (n = 39) patients, respectively. The dose for each patient was appropriately titrated based on efficacy and safety. Response in PV patients was defined based on hematocrit control in the absence of phlebotomy, improvement or elimination of palpable splenomegaly, and normalization of leukocytosis and thrombocytosis. Response in ET patients was defined based on improvement or normalization of WBC and platelet counts and elimination of palpable splenomegaly. 

In patients with PV, 97% achieved hematocrit control to <45% in the absence of phlebotomy, and all patients maintained phlebotomy independence (median follow-up of 15 months, range 8–21). In the 74% of patients who had splenomegaly at baseline, 59% achieved at least a 50% reduction in palpable spleen length or the spleen became non-palpable. Leukocytosis (WBC count >15 × 10^9^/L), present in 47% of patients at baseline, improved (≤15 × 10^9^/L) in 88% and normalized (≤ the upper limit of normal [ULN]) in 63% of patients. Thrombocytosis >600 × 10^9^/L, present in 38% of patients at baseline, improved in 92% (≤ 600 × 10^9^/L) and normalized (≤ ULN) in 69% of patients. Overall, 59% of patients achieved phlebotomy independence, resolution of splenomegaly and normalization of leukocytosis and thrombocytosis. Grade 3 AEs potentially related to study medication included thrombocytopenia (n = 2), and neutropenia, renal tumor, asthenia, viral infection, and atrial flutter (n = 1 each). There were no grade 4 drug-related AEs. Three patients discontinued therapy because of AEs.

In patients with ET, platelet counts normalized (≤ ULN) in 49% of patients after a median of 0.5 months. This response was maintained for a median of 3.5 months; 82% maintained platelet counts <600 × 10^9^/L for a median of 9.8 months. Among patients with baseline platelet counts >1,000 × 10^9^/L (n = 14), 13 experienced a >50% reduction. Normal WBC counts were maintained for a median duration 14.5 months. Palpable spleens resolved in 3 of 4 patients. Grade 3 AEs potentially related to study medication included leukopenia (n = 2), GI disorder (n = 1), and peripheral neuropathy (n = 1). No grade 4 drug-related AEs were reported. Four patients discontinued therapy because of AEs.

Patients with PV and ET demonstrated improvements in symptom scores for itching, night sweats, and bone pain. In addition, 42% of PV and 56% of ET patients had at least a 20% decrease in *JAK2*^V617F^ allele burden. Clinical efficacy was not related to the presence/absence of the *JAK2*^V617F^ mutation or changes in allele burden [107]. There are no published data on the effect of ruxolitinib on thrombotic risk in patients with PV or ET.

### Other Ruxolitinib Trials in MPN

Ruxolitinib is currently in clinical trials in patients with other subtypes of MPN. RESPONSE (Randomized Study of Efficacy and Safety in Polycythemia Vera with JAK Inhibitor INCB018424 Versus Best Available Care; ClinicalTrials.gov number, NCT01243944) is an ongoing global, open-label phase III trial designed to compare the efficacy and safety of ruxolitinib to BAT (1:1) in 200 adult patients with PV who are resistant/refractory to or intolerant of HU (by ELN criteria), require phlebotomy due to inadequate hematocrit control, and exhibit palpable splenomegaly with a two times normal spleen volume. The composite primary efficacy endpoint after 32 weeks of treatment is based on achieving both phlebotomy independence and a reduction in spleen volume. The secondary endpoints are the proportion of patients who maintain the primary endpoint response at 48 weeks from randomization and the proportion of patients achieving complete hematologic remission at 32 weeks. All patients will be treated for 48 weeks to assess safety and response durability. Patients randomized to BAT may be eligible to cross over to receive ruxolitinib after week 32. 

Two clinical trials are investigating use of ruxolitinib in patients with MF and low platelet counts (i.e., <100 × 10^9^/L): CINC424A2201 (ClinicalTrials.gov number, NCT01317875) and INCB018424-258 (ClinicalTrials.gov number, NCT01348490). As clinical experience with ruxolitinib broadens, eventually, this drug will acquire its place in the treatment of landscape of MF and PV. As is the case with every significant advance in therapy, clinical development efforts have already started and will predictably intensify to evaluate ways to optimize ruxolitinib’s use by testing it in combination therapies (e.g., with panobinostat).

## CONCLUSION

The enhanced molecular understanding of MPN has paved the way for the development of clinically and biologically more satisfactory treatments. The current experience with ruxolitinib clearly establishes the dawn of the JAK-targeted era. The drug is generally well tolerated in a wide spectrum of MPN patients, and results in clinically significant rapid and durable reductions in splenomegaly and improvements in disease-related symptoms. Ruxolitinib is currently in clinical trials involving patients with MF and low platelets and patients with PV (phase II completed in PV and ET), and there is enthusiasm to support the development of well-tolerated therapy for all MPN sub-categories, as well as the myeloproliferative variants of myelodysplastic syndromes (the MDS/MPN ‘overlap’ phenotype). The latter group of studies should help assess the potential for reducing the risk of vascular events, which are typically more common in PV and ET, as well as assess ruxolitinib’s activity in MDS/MPN. Furthermore, the recent observation from a large phase III study in MF, COMFORT-I, suggests that there may be a survival benefit with ruxolitinib versus placebo. Clearly, persistence of this survival advantage with longer follow-up would more strongly suggest that inhibition of JAK1 and JAK2 by ruxolitinib can effectively modify the long-term disease progression in patients with MF.

## Figures and Tables

**Table 1.** The 2008 World Health Organization (WHO) Classification of MDS/MPN [2]

**Myeloproliferative Neoplasms (MPN)**
Chronic Myelogenous Leukemia (CML)
Polycythemia Vera (PV)
Essential Thrombocythemia (ET)
Primary Myelofibrosis (PMF)
Chronic Neutrophilic Leukemia
Chronic Eosinophilic Leukemia, Not Otherwise Categorized
Hypereosinophilic Syndrome
Mast Cell Disease
MPN, Unclassifiable
** Myelodysplastic Syndromes (MDS)/MPN**
Chronic Myelomonocytic Leukemia
Juvenile Myelomonocytic Leukemia
Atypical Chronic Myeloid Leukemia
MDS/MPN, Unclassifiable

**Table 2.** Mutations in Myeloproliferative Neoplasms [1, 11, 12]

Associated with Signaling Dysregulation	Associated with Epigenetic Dysregulation	Associated with Leukemic Transformation
JAK2^V617F^ *JAK2* exon 12 MPL LNK CBL NRAS NF1	TET2 EZH2 ASXL1 *PRC2* members DNMT3A	IKZF1 RUNX1 RB TP53 IDH1, -2 DNMT3A

**Table 3.** JAK Inhibitors in Clinical Development for MPN [89]

JAK Kinase Inhibitor	Manufacturer	Main Targets	Clinical Development Phase (MPN)	Clinical Activity	Toxicity
Ruxolitinib	Incyte (US); Novartis (Rest of the world)	JAK1, JAK2	Phase 3 Completed (MF); Approved in US (Intermediate or high-risk MF); 3 (PV), 2 (ET)	Reduction in spleen size; improvement in systemic symptoms, body weight, and performance status; survival advantage versus placebo in MF; normalization of inflammatory cytokine levels in MF; normalization of blood cell counts in ET and PV	Myelosuppression (mainly low platelet counts)
TG101348	Sanofi	JAK2, FLT3, RET	3 (MF) , 2 (PV)	Reduction in spleen size, improvement in systemic symptoms	Myelosuppression, gastrointestinal disturbances
Lestaurtinib	Cephalon	JAK2, FLT3, RET, Trk-A	2 (MF, PV, ET)	Reduction in spleen size, improvement in blood cell count in MF; decrease in blood cell counts in PV and ET	Gastrointestinal disturbances, low platelet counts
CYT387	YM BioSciences	JAK1, JAK2	2 (MF)	Reduction in spleen size, improvement in systemic symptoms, preliminary suggestion of anemia improvement	Myelosuppression
Pacritinib	S*BIO	JAK2, FLT3	2 (MF)	Decrease in spleen size and improvement in symptoms with minimal myelosuppression	Gastrointestinal disturbances
AZD1480	AstraZeneca	JAK1, JAK2	1/2 (MF)	Data not yet available	Data not yet available
LY2784544	Lilly	JAK2	1/2 (ET, PV, MF)	Data not yet available	Data not yet available

Abbreviations: ET, essential thrombocythemia; FLT3, FMS-like tyrosine kinase 3; JAK, Janus kinase; MF, myelofibrosis; MPN, myeloproliferative neoplasm; PV, polycythemia vera. Adapted with permission from the American Cancer Society.

**Table 4.** COMFORT-I: Hematology Laboratory Values (Worst Grade on Study) [103]

	Ruxolitinib, n = 155		Placebo, n = 151	
	All Grades %	Grade 3 or 4 %	All Grades %	Grade 3 or 4 %
Hemoglobin	96.1	45.2	86.8	19.2
Platelets	69.7	12.9	30.5	1.3
Neutrophils	18.7	7.1	4.0	2.0

**Table 5.** COMFORT-II: Hematology Laboratory Values (Worst Grade on Study) [105]

	Ruxolitinib, n = 146		BAT, n = 73	
	Grade 3 %	Grade 4 %	Grade 3 %	Grade 4 %
Hemoglobin	34	8	21	10
Platelets	6	2	4	3

**Fig. (1):**
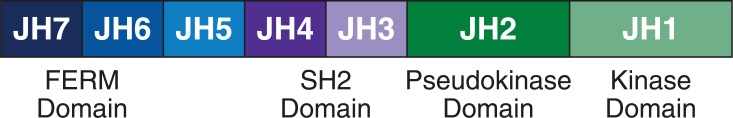
Schematic structure of JAKs. There are seven JAK homology regions (JH) containing the catalytically active kinase domain (JH1), the auto-inhibitory pseudokinase domain (JH2), the SH2 domain (JH3, JH4), and a FERM domain (JH6, JH7) [62]. Reproduced with permission from Wiley-Blackwell.

**Fig. (2):**
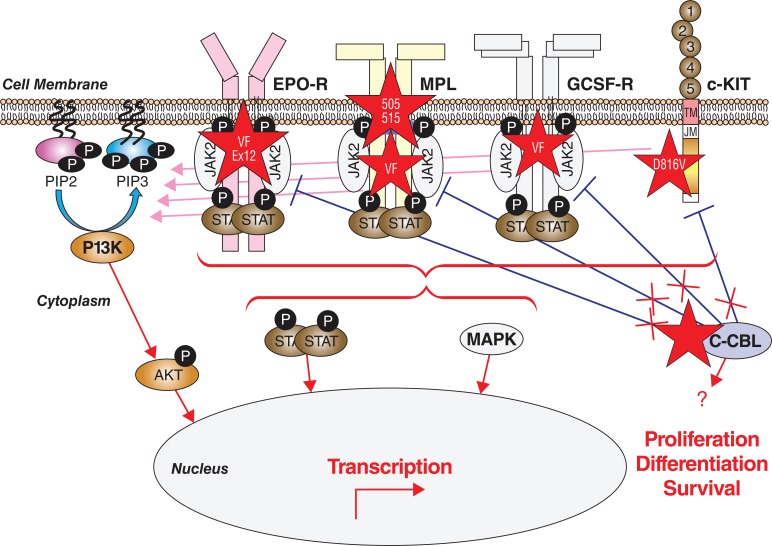
Involvement of the cytokine receptor-tyrosine kinase axis in MPN oncogenesis. The four main myeloid growth factor receptors involved in MPN pathogenesis are represented with their principal downstream signaling event sequences, involving the binding of JAK2, and the phosphorylation of phosphatidyl- inositol-3-kinase (PI3K), protein kinase B (AKT; generally thought to be a cytoplasmic protein), the signal transducers and activators of transcription (STATs), and the mitogen-activated protein kinases (MAPK). The adaptor and E3 ubiquitin ligase c-CBL (casitas B-lineage lymphoma) protein downregulates c-KIT and JAK2 signaling (blue bars). Red stars indicate the oncogenic mutations that occur in MPN resulting in a constitutive or enhanced downstream signaling (red) with eventual modulation of transcription of genes that control and/or modulate cell cycle, proliferation, and apoptosis. Abbreviations: VF, JAK2V617F; Ex12, JAK2 exon 12 mutations; 505 and 515, MPLW515 and MPLS505N mutations, D816V, KITD816V. Several point mutations have been described in c-CBL, resulting in both loss of its inhibitory functions (red crosses) and gain of function properties (red arrow) [71]. Reproduced with permission by the courtesy of International Journal of Hematology.

**Fig. (3):**
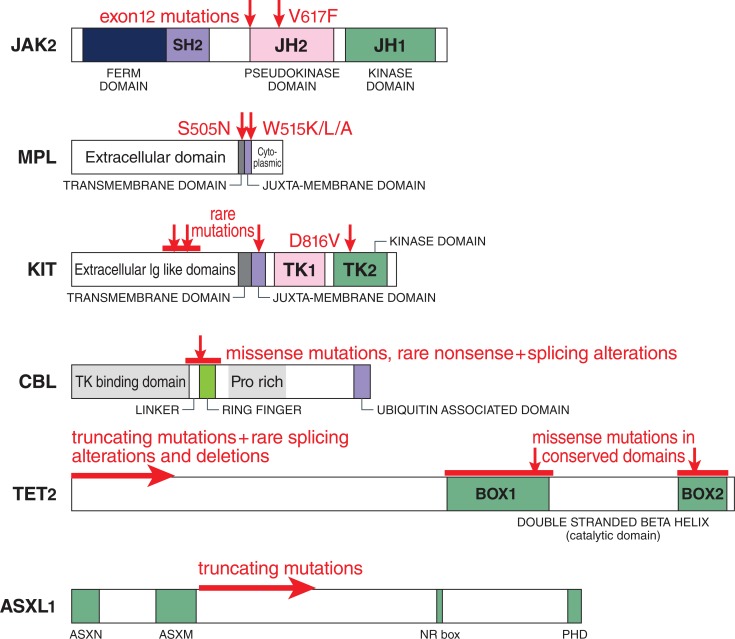
Mutations in MPN. Genes involved in MPN pathogenesis are represented in a schematic linear fashion with their principal functional or conserved domains. Molecular defects are shown in red. Point mutations are indicated by vertical arrows, with horizontal bars spanning the domains where multiple mutations have been identified. Horizontal arrows indicate truncating mutations that may occur anywhere in the downstream coding sequence. Abbreviations: SH, Src homology; JH, JAK homology; Ig, immunoglobulin; TK, tyrosine kinase; Pro, proline; ASXN and ASXM, ASX conserved domains; NR, nuclear receptor; PHD, plant homeodomain [71]. Reproduced with permission by the courtesy of International Journal of Hematology.

**Fig. (4):**
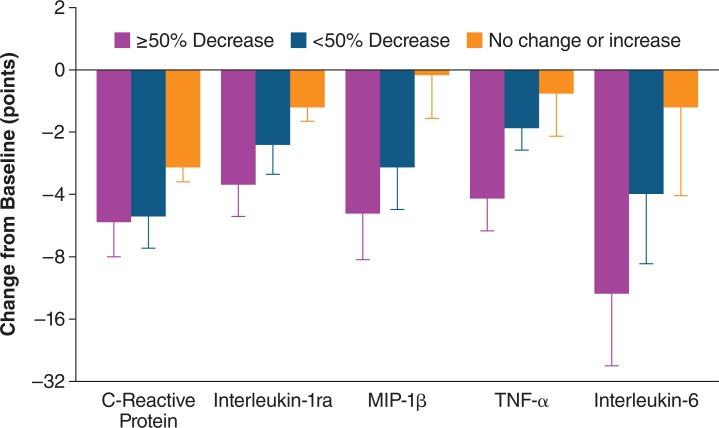
Effect of ruxolitinib treatment on cytokine levels. Changes in selected cytokines and C-reactive protein (CRP), an acute-phase reactant and a marker for inflammation, are shown in MF patients with a 50% or greater decrease, those with less than a 50% decrease, and those with no change or an increase in the composite symptom score after 6 cycles (months) of ruxolitinib treatment as compared with baseline (ClinicalTrials.gov number, NCT00509899) [101]. Reproduced with permission from the Massachusetts Medical Society.

**Fig. (5):**
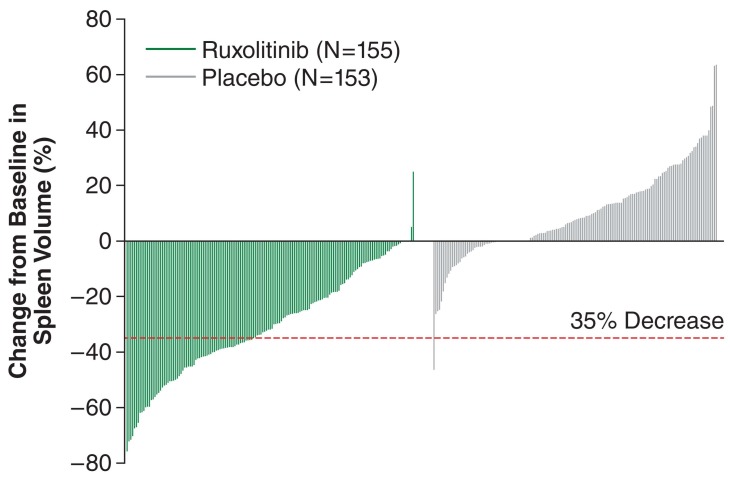
Percent change from baseline in spleen volume at week 24 or last observation for each patient. A significantly larger proportion of patients in the ruxolitinib group achieved a 35% or greater reduction in spleen volume from baseline compared to placebo [103]. Reproduced with permission from the Massachusetts Medical Society.

**Fig. (6):**
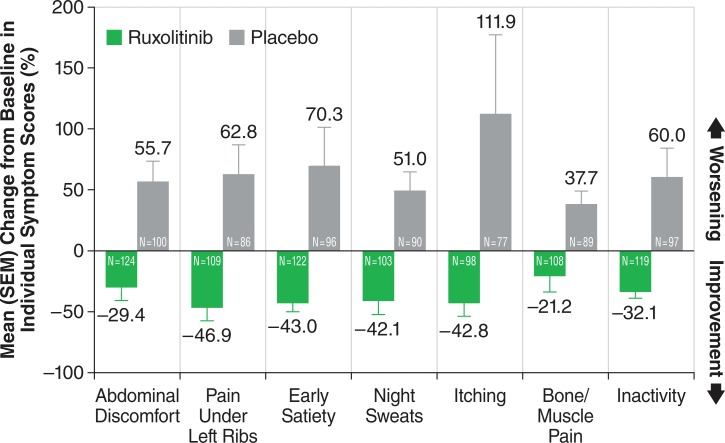
Mean percent change in individual symptom scores at week 24. At week 24, individual symptom scores improved in the ruxolitinib group (decrease from baseline), while scores worsened in the placebo group (increase in score from baseline) (all P <0.01) (reproduced with permission from NEJM) [103]. Reproduced with permission from the Massachusetts Medical Society.

## References

[1] Tefferi A (2010). Novel mutations and their functional and clinical relevance in myeloproliferative neoplasms: JAK2 MPL TET2 ASXL1 CBL IDH and IKZF1. Leukemia.

[2] Tefferi A, Vardiman JW (2008). Classification and diagnosis of myeloproliferative neoplasms: the 2008 World Health Organization criteria and point-of-care diagnostic algorithms. Leukemia.

[3] Tefferi A, Thiele J, Orazi A, Kvasnicka HM, Barbui T, Hanson CA, Barosi G, Verstovsek S, Birgegard G, Mesa R, Reilly JT, Gisslinger H, Vannucchi AM, Cervantes F, Finazzi G, Hoffman R, Gilliland DG, Bloomfield CD, Vardiman JW (2007). Proposals and rationale for revision of the World Health Organization diagnostic criteria for polycythemia vera essential thrombocythemia, and primary myelofibrosis: recommendations from an ad hoc international expert panel. Blood.

[4] Baxter EJ, Scott LM, Campbell PJ, East C, Fourouclas N, Swanton S, Vassiliou GS, Bench AJ, Boyd EM, Curtin N, Scott MA, Erber WN, Green AR (2005). Acquired mutation of the tyrosine kinase JAK2 in human myeloproliferative disorders. Lancet.

[5] James C, Ugo V, Le Couedic JP, Staerk J, Delhommeau F, Lacout C, Garçon L, Raslova H, Berger R, Bennaceur-Griscelli A, Villeval JL, Constantinescu SN, Casadevall N, Vainchenker W (2005). A unique clonal JAK2 mutation leading to constitutive signalling causes polycythaemia vera. Nature.

[6] Kralovics R, Passamonti F, Buser AS, Teo SS, Tiedt R, Passweg JR, Tichelli A, Cazzola M, Skoda RC (2005). A gain-of-function mutation of JAK2 in myeloproliferative disorders. N. Engl. J. Med.

[7] Levine RL, Wadleigh M, Cools J, Ebert BL, Wernig G, Huntly BJ, Boggon TJ, Wlodarska I, Clark JJ, Moore S, Adelsperger J, Koo S, Lee JC, Gabriel S, Mercher T, D'Andrea A, Fröhling S, Döhner K, Marynen P, Vandenberghe P, Mesa RA, Tefferi A, Griffin JD, Eck MJ, Sellers WR, Meyerson M, Golub TR, Lee SJ, Gilliland DG (2005). Activating mutation in the tyrosine kinase JAK2 in polycythemia vera, essential thrombocythemia, and myeloid metaplasia with myelofibrosis. Cancer Cell.

[8] Zhao R, Xing S, Li Z, Fu X, Li Q, Krantz SB, Zhao ZJ (2005). Identification of an acquired JAK2 mutation in polycythemia vera. J. Biol. Chem.

[9] Tefferi A, Skoda R, Vardiman JW (2009). Myeloproliferative neoplasms: contemporary diagnosis using histology and genetics. Nat. Rev. Clin. Oncol.

[10] Verstovsek S (2010). Therapeutic potential of JAK2 inhibitors. Hematology Am. Soc. Hematol. Educ. Program.

[11] Passamonti F, Maffioli M, Caramazza D, Cazzola M (2011). Myeloproliferative neoplasms: From JAK2 mutations discovery to JAK2 inhibitor therapies. Oncotarget.

[12] Walter MJ, Ding L, Shen D, Shao J, Grillot M, McLellan M, Fulton R, Schmidt H, Kalicki-Veizer J, O'Laughlin M, Kandoth C, Baty J, Westervelt P, DiPersio JF, Mardis ER, Wilson RK, Ley TJ, Graubert TA (2011). Recurrent DNMT3A mutations in patients with myelodysplastic syndromes. Leukemia.

[13] Berlin NI (1975). Diagnosis and classification of the polycythemias. Semin. Hematol.

[14] Michiels JJ, De Raeve H, Berneman Z (2006). The 2001 World Health Organization and updated European clinical and pathological criteria for the diagnosis classification, and staging of the.Philadelphia chromosome-negative chronic myeloproliferative disorders. Semin. Thromb. Hemost.

[15] Passamonti F, Rumi E, Caramella M, Elena C, Arcaini L, Boveri E, Del Curto C, Pietra D, Vanelli L, Bernasconi P, Pascutto C, Cazzola M, Morra E, Lazzarino M (2008). A dynamic prognostic model to predict survival in post-polycythemia vera myelofibrosis. Blood.

[16] Passamonti F, Cervantes F, Vannucchi AM, Morra E, Rumi E, Pereira A, Guglielmelli P, Pungolino E, Caramella M, Maffioli M, Pascutto C, Lazzarino M, Cazzola M, Tefferi A (2010). A dynamic prognostic model to predict survival in primary myelofibrosis: a study by the IWG-MRT (International Working Group for Myeloproliferative Neoplasms Research and Treatment). Blood.

[17] Budde U, Scharf RE, Franke P, Hartmann-Budde K, Dent J, Ruggeri ZM (1993). Elevated platelet count as a cause of abnormal von Willebrand factor multimer distribution in plasma. Blood.

[18] Finazzi G, Barbui T (2005). Risk-adapted therapy in essential thrombocythemia and polycythemia vera. Blood Rev.

[19] Barbui T, Barosi G, Birgegard G, Cervantes F, Finazzi G, Griesshammer M, Harrison C, Hasselbalch HC, Hehlmann R, Hoffman R, Kiladjian JJ, Kröger N, Mesa R, McMullin MF, Pardanani A, Passamonti F, Vannucchi AM, Reiter A, Silver RT, Verstovsek S, Tefferi A (2011). European LeukemiaNet. Philadelphia-negative classical myeloproliferative neoplasms: critical concepts and management recommendations from European LeukemiaNet. J. Clin. Oncol.

[20] Tefferi A (2011). Annual Clinical Updates in Hematological Malignancies: a continuing medical education series: polycythemia vera and essential thrombocythemia: 2011 update on diagnosis, risk-stratification, and man-agement. Am. J. Hematol.

[21] Barosi G, Birgegard G, Finazzi G, Griesshammer M, Harrison C, Hasselbalch HC, Kiladjian JJ, Lengfelder E, McMullin MF, Passamonti F, Reilly J T, Vannucchi AM, Barbui T (2009). Response criteria for essential thrombocythemia and polycythemia vera: result of a European LeukemiaNet consensus conference. Blood.

[22] Pearson TC, Weatherly-Mein G (1978). Vascular occlusive episodes and venous hae¬matocrit in primary proliferative polycythaemia. Lancet.

[23] Finazzi G, Barbui T (2007). How I treat patients with polycythemia vera. Blood.

[24] Menagé HD, Norris PG, Hawk JL, Graves MW (1993). The efficacy of psoralen photochemotherapy in the treatment of aquagenic pruritus. Br. J. Dermatol.

[25] Finelli C, Gugliotta L, Gamberi B, Vianelli N, Visani G, Tura S (1993). Relief of intractable pruritus in polycythemia vera with recombinant interferon alfa. Am. J. Hematol.

[26] Squizzato A, Romualdi E, Middeldorp S (2008). Antiplatelet drugs for polycythae¬mia vera and essential thrombocythaemia. Cochrane Database Syst. Rev.

[27] Landolfi R, Marchioli R, Kutti  J, Gisslinger H, Tognoni G, Patrono P, Barbui T (2010). the European Collaboration on Low-Dose Aspirin in Polycythemia Vera Investigators.Efficacy and safety of low-dose aspirin in polycythemia vera. N. Engl. J. Med.

[28] Finazzi G, Caruso V, Marchioli R, Capnist G, Chisesi T, Finelli C, Gugliotta L, Landolfi R, Kutti J, Gisslinger H, Marilus R, Patrono C, Pogliani EM, Randi ML, Villegas A, Tognoni G, Barbui T (2005). Acute leukemia in polycythemia vera: an analysis of 1638 patients enrolled in a prospective observational study. Blood.

[29] Najean Y, Rain JD (1997). Treatment of polycythemia vera: the use of hydroxy-urea and pipobroman in 292 patients under the age of 65 years. Blood.

[30] Kiladjian JJ, Rain JD, Bernard JF, Briere J, Chomienne C, Fenaux P (2006). Long-term incidence of hematological evolution in three French prospective studies of hydroxyurea and pipobroman in polycythemia vera and essential thrombocythe¬mia. Semin. Thromb. Hemost.

[31] Kiladjian JJ, Chevret S, Dosquet C, Chomienne C, Rain JD (2011). Treatment of polycythemia vera with hydroxyurea and pipobroman: final results of a randomized trial initiated in 1980. J. Clin. Oncol.

[32] Hasselbalch HC, Kiladjian JJ, Silver RT (2011). Interferon-alpha in the treatment of Philadelphia-negative chronic myeloproliferative neoplasms. J. Clin. Oncol.

[33] Kiladjian JJ, Cassinat B, Chevret S, Turlure P, Cambier N, Roussel M, Bellucci S, Grandchamp B, Chomienne C, Fenaux P (2008). Pegylated interferon-alfa-2a induces complete hematologic and molecular responses with low toxicity in polycythemia vera. Blood.

[34] Fabris F, Randi ML (2009). Essential thrombocythemia: past and present. Intern. Emerg. Med.

[35] Mesa RA, Niblack J, Wadleigh M, Verstovsek S, Camoriano J, Bar-nes S, Tan AD, Atherton PJ, Sloan JA, Tefferi A (2007). The burden of fatigue and quality of life in myeloproliferative disorders (MPDs): an in-ternational Internet-based survey of 1179 MPD patients. Cancer.

[36] Jensen MK, de Nully Brown P, Lund BV, Nielsen OJ, Hasselbalch HC (2001). Increased circulating platelet-leukocyte aggregates in myeloproliferative disorders is correlated to previous thrombosis, platelet activation and platelet count. Eur. J. Haematol.

[37] Arellano-Rodrigo E, Alvarez-Larran A, Reverter JC, Villamor N, Colomer D, Cervantes F (2006). Increased platelet and leukocyte activation as contributing mechanisms for thrombosis in essential thrombocythemia and correlation with the JAK2 mutational status. Haematologica.

[38] Cortelazzo S, Finazzi G, Ruggeri M, Vestri O, Galli M, Rodeghiero F, Barbui T (1995). Hydroxyurea for patients with essential thrombocythemia and a high risk of thrombosis. N. Engl. J. Med.

[39] Spivak JL, Barosi G, Tognoni G, Barbui T, Finazzi G, Marchioli R, Marchetti M (2003). Chronic myeloproliferative disorders. Hematol. Am. Soc. He-matol. Educ. Prog.

[40] Quintás-Cardama A, Kantarjian H, Manshouri T, Luthra R, Estrov Z, Pierce S, Richie MA, Borthakur G, Konopleva M, Cortes J, Verstovsek S (2009). Pegylated interferon alfa-2a yields high rates of hematologic and molecular response in patients with advanced essential thrombocythemia and polycythemia vera. J. Clin. Oncol.

[41] Harrison CN, Campbell PJ, Buck G, Wheatley K, East CL, Bareford D, Wilkins BS, van der Walt JD, Reilly JT, Grigg AP, Revell P, Woodcock BE, Green AR (2005). Hydroxyurea compared with anagrelide in high-risk essential thrombocythemia. N. Engl. J. Med.

[42] van Genderen PJ, Mulder PG, Waleboer M, van de Moesdijk D, Michiels JJ (1997). Prevention and treatment of thrombotic complications in essential thrombocy¬thaemia: efficacy and safety of aspirin. Br. J. Haematol.

[43] Zhan H, Spivak JL (2009). The diagnosis and management of polycythemia vera, essential thrombocythemia, and primary myelofibrosis in the JAK2 V617F era. Clin. Adv. Hematol. Oncol.

[44] Cervantes F, Dupriez B, Pereira A, Passamonti F, Reilly JT, Morra E, Vannucchi AM, Mesa RA, Demory JL, Barosi G, Rumi E, Tefferi A (2009). A new prognostic scoring system for primary myelofibrosis based on a study of the International Working Group for Myelofibrosis Research and Treatment. Blood.

[45] Tefferi A (2008). Management of myeloproliferative neoplasms and the promise of targeted therapy.

[46] Gangat N, Caramazza D, Vaidya R, George G, Begna K, Schwager S, Hanson C, Wu W, Pardanani A, Cervantes F, Passamonti F, Tefferi A (2011). DIPSS plus: a refined Dynamic International Prognostic Scoring System for primary myelofibrosis that incorporates prognostic information from karyotype, platelet count, and transfusion status. J. Clin. Oncol.

[47] Tefferi A (2011). How I treat myelofibrosis. Blood.

[48] Tefferi A (2010). Allogeneic hematopoietic cell transplantation versus drugs in myelofibrosis: the risk-benefit balancing act. Bone Marrow Transplant.

[49] Ballen KK, Shrestha S, Sobocinski KA, Zhang MJ, Bashey A, Bolwell BJ, Cervantes F, Devine SM, Gale RP, Gupta V, Hahn TE, Hogan WJ, Kröger N, Litzow MR, Marks DI, Maziarz RT, McCarthy PL, Schiller G, Schouten HC, Roy V, Wiernik PH, Horowitz MM, Giralt SA, Arora M (2010). Outcome of transplantation for myelofibrosis. Biol. Blood Marrow Transplant.

[50] Patriarca F, Bacigalupo A, Sperotto A, Isola M, Soldano F, Bruno B, van Lint MT, Iori AP, Santarone S, Porretto F, Pioltelli P, Visani G, Iacopino P, Fanin R, Bosi A (2008). GITMO Allogeneic hematopoietic stem cell transplantation in myelofibrosis: the 20-year experience of the Gruppo Italiano Trapianto di Midollo Osseo (GITMO). Haematologica.

[51] Rondelli D, Goldberg JD, Marchioli R, Isola L, Shore TB, Prchal JT, Bacigalupo A, Rambaldi A, Klisovic RB, Gupta V, Andreasson B, Demakos EP, Price LS, Scarano M, Wetzler M, Vannucchi AM, Najfeld V, Barosi G, Silverman LR, Hoffman R (2011). Results of phase II clinical trial MPD-RC 101: allogeneic hematopoietic stem cell transplantation conditioned with fludarabine/melphalan in patients with myelofibrosis. Blood [ASH Annual Meeting Abstracts].

[52] Arana-Yi C, Quintás-Cardama A, Giles F, Thomas D, Carrasco-Yalan A, Cortes J, Kantarjian H, Verstovsek S (2006). Advances in the therapy of chronic idiopathic myelofibrosis. Oncologist.

[53] Silver RT, Vandris K, Goldman J (2011). Recombinant interferon-a may retard the progression of early primary myelofibrosis: a preliminary report. Blood.

[54] Vainchenker W, Dusa A, Constantinescu SN (2008). JAKs in pathology: role of Janus kinases in hematopoietic malignancies and immunodeficiencies. Semin. Cell Dev. Biol.

[55] Wilks AF (1989). Two putative protein-tyrosine kinases identified by application of the polymerase chain reaction. Proc. Natl. Acad. Sci. U.S.A.

[56] Wilks AF, Harpur AG, Kurban RR, Ralph SJ, Zurcher G, Ziemiecki A (1991). Two novel protein-tyrosine kinases, each with a second phosphotransferase related catalytic domain, define a new class of protein kinase. Mol. Cell Biol.

[57] Watowich SS, Wu H, Socolovsky M, Klingmuller U, Constantinescu SN, Lodish HF (1996). Cytokine receptor signal transduction and the control of hematopoietic cell development. Annu. Rev. Cell Dev. Biol.

[58] Firmbach-Kraft I, Byers M, Shows T, Dalla-Favera R, Krolewski JJ (1990). tyk2, prototype of a novel class of non-receptor tyrosine kinase genes. Oncogene.

[59] Riedy MC, Dutra AS, Blake TB, Modi W, Lal BK, Davis J, Bosse A, O'Shea JJ, Johnston JA (1996). Genomic sequence organization and chromosomal localization of human JAK3. Genomics.

[60] Leonard WJ, O’Shea JJ (1998). Jaks and STATs: biological implications. Annu. Rev. Immunol.

[61] Radtke S, Haan S, Jörissen A, Hermanns HM, Diefenbach S, Smyczek T, Schmitz-Vandeleur H, Heinrich PC, Behrmann I, Haan C (2005). The Jak1 SH2 domain does not fulfill a classical SH2 function in Jak/STAT signaling but plays a structural role for receptor interaction and up-regulation of receptor surface expression. J. Biol. Chem.

[62] Ghoreschi K, Laurence A, O'Shea JJ (2009). Janus kinases in immune cell signaling. Immunol. Rev.

[63] Sandberg EM, Wallace TA, Godeny MD, VonDerLinden D, Sayeski PP (2004). Jak2 tyrosine kinase: a true jak of all trades?. Cell Biochem. Biophys.

[64] Lohi O, Lehto VP (2001). STAM/EAST/Hbp adapter proteins – integrators of signalling pathways. FEBS Lett.

[65] Rawlings JS, Rosler KM, Harrison DA (2004). The JAK/STAT signaling pathway. J. Cell Sci.

[66] O’Brien KB, O’Shea JJ, Carter-Su C (2002). SH2-B family members differentially regulate JAK family tyrosine kinases. J. Biol. Chem.

[67] Yoshimura A, Naka T, Kubo M (2007). SOCS.proteins cytokine signalling and immune regulation. Nat. Rev. Immunol.

[68] Johnston JA, O’Shea JJ (2003). Matching SOCS with function. Nat. Immunol.

[69] Oppliger Leibundgut E, Horn MP, Brunold C, Pfanner-Meyer B, Marti D, Hirsiger H, Tobler A, Zwicky C (2006). Hematopoietic and endothelial progenitor cell trafficking in patients with myeloproliferative diseases. Haematologica.

[70] Tiedt R, Hao-Shen H, Sobas MA, Looser R, Dirnhofer S, Schwaller J, Skoda RC (2008). Ratio of mutant JAK2-V617F to wild type JAK2 determines the MPD phenotypes in transgenic mice. Blood.

[71] Delhommeau F, Jeziorowska D, Marzac C, Casadevall N (2010). Molecular aspects of myeloproliferative neoplasms. Int. J. Hematol.

[72] Dawson MA, Bannister AJ, Göttgens B, Foster SD, Bartke T, Green AR, Kouzarides T (2009). JAK2 phosphorylates histone H3Y41 and excludes HPI alpha from chromatin. Nature.

[73] Butcher CM, Hahn U, To LB, Gecz J, Wilkins EJ, Scott HS, Bardy PG, D'Andrea RJ (2008). Two novel JAK2 exon 12 mutations in JAK2V617F-negative polycythaemia vera patients. Leukemia.

[74] Abdel-Wahab O, Pardanani A, Bernard OA, Finazzi G, Crispino JD, Gisslinger H, Kralovics R, Odenike O, Bhalla K, Guptas V, Barosi G, Gotlib J, Guglielmelli P, Kiladjian J-J, Noel P, Cazzola M, Vannucchi AM, Hoffman R, Barbui T, Thiele J, Van Etten RA, Mughal T, Tefferi A Unraveling the genetic underpinnings of myeloproliferative neoplasms and understanding their effect on disease course and response to therapy: Proceedings from the 6th International Post-ASH Symposium. Am. J. Hematol.

[75] Pikman Y, Lee  BH, Mercher T, McDowell E, Ebert BL, Gozo M, Cuker A, Wernig G, Moore S, Galinsky I, DeAngelo DJ, Clark JJ, Lee SJ, Golub TR, Wadleigh M, Gilliland DG, Levine RL (2006). MPLW515L is a novel somatic activating mutation in myelofibrosis with myeloid metaplasia. PLoS Med.

[76] Abdel-Wahab O, Pardanani A, Rampal R, Lasho TL, Levine RL, Tefferi A (2011). DNMT3A mutational analysis in primary myelofibrosis, chronic myelomonocytic leukemia and advanced phases of myeloproliferative neoplasms. Leukemia.

[77] Stegelmann F, Bullinger L, Schlenk RF, Paschka P, Griesshammer M, Blersch C, Kuhn S, Schauer S, Döhner H, Döhner K (2011). DNMT3A mutations in myeloproliferative neoplasms. Leukemia.

[78] Ernst T, Chase AJ, Score J, Hidalgo-Curtis CE, Bryant C, Jones AV, Waghorn K, Zoi K, Ross FM, Reiter A, Hochhaus A, Drexler HG, Duncombe A, Cervantes F, Oscier D, Boultwood J, Grand FH, Cross NCP (2010). Inactivating mutations of the histone methyltransferase gene *EZH2* in myeloid disorders. Nat. Genet.

[79] Brecqueville M, Cervera N, Adélaïde J, Rey J, Carbuccia N, Chaffanet M, Mozziconacci MJ, Vey N, Birnbaum D, Gelsi-Boyer V, Mruati A (2011). Mutations and deletions of the SUZ12 polycomb gene in myeloproliferative neoplasms. Blood Cancer J.

[80] Score J, Hidalgo-Curtis C, Jones AV, Winkelmann N, Skinner A, Ward D, Zoi K, Ernst T, Stegelmann F, Döhner K, Chase A, Cross NC (2012). Inactivation of polycomb repressive complex 2 components in myeloproliferative and myelodysplastic/myeloproliferative neoplasms. Blood.

[81] Puda A, Milosevic JD, Berg T, Klampfl T, Harutyunyan AS, Gisslinger B, Rumi E, Pietra D, Malcovati L, Elena C, Doubek M, Steurer M, Tosic N, Pavlovic S, Guglielmelli P, Pieri L, Vannucchi AM, Gisslinger H, Cazzola M, Kralovics R Frequent deletions of JARID2 in leukemic transformation of chronic myeloid malignancies. Am. J. Hematol.

[82] Vainchenker W, Delhommeau F, Constantinescu SN, Bernard ON (2011). New mutations and pathogenesis of myeloproliferative neoplasms. Blood.

[83] Sanada M, Suzuki T, Shih LY, Otsu M, Kato M, Yamazaki S, Ta-mura A, Honda H, Sakata-Yanagimoto M, Kumano K, Oda H, Yamagata T, Takita J, Gotoh N, Nakazaki K, Kawamata N, Onodera M, Nobuyoshi M, Hayashi Y, Harada H, Kurokawa M, Chiba S, Mori H, Ozawa K, Omine M, Hirai H, Nakauchi H, Koeffler HP, Ogawa S (2009). Gain-of-function of mutated C-CBL tumour suppressor in myeloid neoplasms. Nature.

[84] Abdel-Wahab O, Manshouri T, Patel J, Harris K, Yao J, Hedvat C, Heguy A, Bueso-Ramos C, Kantarjian H, Levine RL, Verstovsek S (2010). Genetic analysis of transforming events that convert chronic myeloproliferative neoplasms to leukemias. Cancer Res.

[85] Couronné L, Lippert E, Andrieux J, Kosmider O, Radford-Weiss I, Penther D, Dastugue N, Mugneret F, Lafage M, Gachard N, Nadal N, Bernard OA, Nguyen-Khac F (2010). Analyses of TET2 mutations in post-myeloproliferative neoplasm acute myeloid leukemias. Leukemia.

[86] Tefferi A, Levine RL, Lim KH, Abdel-Wahab O, Lasho TL, Patel J, Finke CM, Mullally A, Li CY, Pardanani A, Gilliland DG (2009). Frequent TET2 mutations in systemic mastocytosis: clinical, KITD816V and FIP1L1-PDGFRA correlates. Leukemia.

[87] Bruchova H, Merkerova M, Prchal JT (2008). Aberrant expression of microRNA in polycythemia vera. Haematologica.

[88] Quintas-Cardama A, Kantarjian H, Cortes J, Verstovsek S (2011). Janus kinase inhibitors for the treatment of myeloproliferative neoplasias and beyond. Nat. Rev. Drug Discov.

[89] Quintas-Cardama A, Verstovsek S Spleen deflation and beyond: The pros and cons of Janus kinase 2 inhibitor therapy for patients with myeloproliferative neoplasms. Cancer.

[90] Pardanani A, Gotlib JR, Jamieson C, Cortes JE, Talpaz M, Stone RM, Silverman MH, Gilliang DG, Shorr J, Tefferi T (2011). Safety and efficacy of TG101348 a selective.JAK2 inhibitor in myelofibrosis. J. Clin. Oncol.

[91] Knapper S, Burnett AK, Littlewood T, Kell WJ, Agrawal S, Chopra R, Clark R, Levis MJ, Small D (2006). A phase 2 trial of the FLT3 inhibitor lestaurtinib (CEP701) as first-line treatment for older patients with acute myeloid leukemia not considered fit for intensive chemotherapy. Blood.

[92] Smith BD, Levis M, Beran M, Giles F, Kantarjian H, Berg K, Mur-phy KM, Dauses T, Allebach J, Small D (2004). Single-agent CEP-701.novel FLT3 inhibitor shows biologic and clinical activity in patients with relapsed or refractory acute myeloid leukemia. Blood.

[93] Pardanani A, Lasho T, Smith G, Burns CJ, Fantino E, Tefferi A (2009). CYT387 a selective JAK1/JAK2 inhibitor: in vitro assessment of kinase selectivity and preclinical studies using cell lines and primary cells from polycythemia vera patients. Leukemia.

[94] Tyner JW, Bumm TG, Deininger J, Wood L, Aichberger KJ, Loriaux MM, Druker BJ, Burns CJ, Fantino E, Deininger MW (2010). CYT387 a novel JAK2 inhibitor induces hematologic responses and normalizes inflammatory cytokines in murine myeloproliferative neoplasms. Blood.

[95] Pardanani A, George G, Lasho T, Hogan WJ, Litzow MR, Begna K, Hanson CA, Fida R, Burns C, Smith GD, Tefferi A (2010). A phase I/II study of CYT387, an oral JAK-1/2 inhibitor in myelofibrosis: significant response rates in anemia splenomegaly and constitutional symptoms. Blood [ASH Annual Meeting Abstracts].

[96] Komrokji RS, Wadleigh M, Seymore JF, Roberts AW, Bik To L, Zhu HJ, Mesa RA (2011). Results of a phase 2 study of Pacritinib (SB1518): a novel oral JAK2 inhibitor, in patients with primary, post-polycythemia vera, and post-essential thrombocythemia myelofibrosis. Blood [ASH Annual Meeting Abstracts].

[97] Hedvat M, Huszar D, Herrmann A, Gozgit JM, Schroeder A, Sheehy A, Buettner R, Proia D, Kowolik CM, Xin H, Armstrong B, Bebernitz G, Weng S, Wang L, Ye M, McEachern K, Chen H, Morosini D, Bell K, Alimzhanov M, Ioannidis S, McCoon P, Cao ZA, Yu H, Jove R, Zinda M (2009). The JAK2 inhibitor AZD1480 potently blocks Stat3 signaling and oncogenesis in solid tumors. Cancer Cell.

[98] Quintás-Cardama A, Vaddi K, Liu P, Manshouri T, Li J, Scherle PA, Caulder E, Wen X, Li Y, Waeltz P, Rupar M, Burn T, Lo Y, Kelley J, Covington M, Shepard S, Rodgers JD, Haley P, Kantarjian H, Fridman JS, Verstovsek S (2010). Preclinical characterization of the selective JAK1/2 inhibitor INCB018424: therapeutic implications for the treatment of myeloproliferative neoplasms. Blood.

[99] Shilling AD, Nedza FM, Emm T, Diamond S, McKeever E, Punwani N, Williams W, Arvanitis A, Galya LG, Li M, Shepard S, Rodgers J, Yue TY, Yeleswaram S (2010). Metabolism, excretion, and pharmacokinetics of [14C]INCB018424.A selective Janus tyrosine kinase 1/2 inhibitor in humans. Drug Metab. Dispos.

[100] Shi JG, Chen X, Emm T, Scherle PA, McGee RF, Lo Y, Landman RR, McKeever EG, Punwani NG, Williams WV, Yeleswaram S The effect of CYP3A4 inhibition or induction on the pharmacokinetics and pharmacodynamics of orally administered ruxolitinib in healthy volunteers. J. Clin. Pharm.

[101] Verstovsek S, Kantarjian H, Mesa RA, Pardanani AD, Cortes-Franco J, Thomas DA, Estrov Z, Fridman JS, Bradley EC, Erickson-Viitanen S, Vaddi K, Levy R, Tefferi A (2010). Safety and efficacy of INCB018424, a JAK1 and JAK2 inhibitor, in myelofibrosis. N. Engl. J. Med.

[102] Mesa RA, Verstovsek S, Kantarjian HM, Pardanani AD, Friedman S, Newton R, Erickson-Viitanen S, Hunter D, Redman J, Yeleswaram S, Bradley E, Tefferi A (2008). INCB018424 a selective JAK1/2 inhibitor significantly improves the compromised nutritional status and frank cachexia in patients with myelofibrosis. (MF). Blood [ASH Annual Meeting Abstracts].

[103] Verstovsek S, Mesa RA, Gotlib J, Levy RS, Gupta V, DiPersio JF, Catalano JV, Deininger M, Miller C, Silver RT, Talpaz M, Winton EF, Harvey JH, Arcasoy MO, Hexner E, Lyons RM, Paquette R, Raza A, Vaddi K, Erickson-Viitanen S, Koumenis IL, Sun W, Sandor V, Kantarjian HM (2012). A double-blind placebo-controlled trial of ruxolitinib for myelofibrosis. N. Engl. J. Med.

[104] Mesa R, Gotlib J, Gupta V, DiPersio J, Catalano J, Deininger M, Sheilds A, Miller C, Silver R, Talpaz M, Winton E, Harvey J, Hare T, Erickson-Viitanen S, Sun W, Sandor V, Levy R, Kantarjian H, Verstovsek S (2011). COMFORT-I Investigators. Associations between improvements
in myelofibrosis (MF) symptoms and quality of life measures with
splenomegaly reduction in COMFORT-I: a randomized, double-blind, phase
III trial of the JAK1 and JAK2 inhibitor ruxolitinib versus placebo in patients
with MF. Blood [ASH Annual Meeting Abstracts].

[105] Harrison C, Kiladjian JJ, Al-Ali HK, Gisslinger H, Waltzman R, Stalbovskaya V, McQuitty M, Hunter DS, Levy RS, Knoops L, Cervantes F, Vannucchi AM, Barbui T, Barosi G (2012). JAK inhibition with ruxolitinib vs best available therapy in myelofibrosis. N. Engl. J. Med.

[106] Verstovsek S, Deeg HJ, Odenike O, Zhu J, Kantarjian H, Estrov Z, Scott BL, Thomas DA (2010). Phase 1/2 study of SB1518, a novel JAK2/FLT3 inhibitor, in the treatment of primary myelofibrosis. Blood [ASH Annual Meeting Abstracts].

[107] Verstovsek S, Passamonti F, Rambaldi A, Barosi G, Rosen PJ, Levy R, Bradley E, Garrett W, Vaddi K, Contel N, Sandor V, Huber RM, Schacter LP, Rumi E, Gattoni E, Antonioli E, Pieri L, Cazzola M, Kantarjian H, Barbui T, Vannucchi AM (2010). Durable responses with the JAK1/ JAK2 inhibitor, INCB018424, in patients with polycythemia vera (PV) and essential thrombocythemia (ET) refractory or intolerant to hydroxyurea (HU). Blood [ASH Annual Meeting Abstracts].

